# Cellular origins and genetic landscape of cutaneous gamma delta T cell lymphomas

**DOI:** 10.1038/s41467-020-15572-7

**Published:** 2020-04-14

**Authors:** Jay Daniels, Peter G. Doukas, Maria E. Martinez Escala, Kimberly G. Ringbloom, David J. H. Shih, Jingyi Yang, Kyle Tegtmeyer, Joonhee Park, Jane J. Thomas, Mehmet E. Selli, Can Altunbulakli, Ragul Gowthaman, Samuel H. Mo, Balaji Jothishankar, David R. Pease, Barbara Pro, Farah R. Abdulla, Christopher Shea, Nidhi Sahni, Alejandro A. Gru, Brian G. Pierce, Abner Louissaint, Joan Guitart, Jaehyuk Choi

**Affiliations:** 1 Department of Dermatology, Northwestern University Feinberg School of Medicine, Chicago, IL USA; 2 Department of Biochemistry and Molecular Genetics, Northwestern University Feinberg School of Medicine, Chicago, IL USA; 3 Department of Systems Biology, The University of Texas MD Anderson Cancer Center, Houston, TX USA; 4 University of Maryland Institute for Bioscience and Biotechnology Research, Rockville, MD USA; 5 Department of Cell Biology and Molecular Genetics, University of Maryland, College Park, MD USA; 6 University of Illinois College of Medicine, Chicago, IL USA; 7 Department of Medicine, Section of Dermatology, University of Chicago Pritzker School of Medicine, Chicago, IL USA; 8 Division of Hematology/Oncology, Northwestern University Feinberg School of Medicine, Chicago, IL USA; 9 Division of Dermatology, City of Hope Comprehensive Cancer Center, Duarte, CA USA; 10 Department of Epigenetics and Molecular Carcinogenesis, The University of Texas MD Anderson Cancer Center, Smithville, TX USA; 11 Department of Bioinformatics and Computational Biology, The University of Texas MD Anderson Cancer Center, Houston, TX USA; 12 Program in Quantitative and Computational Biosciences, Baylor College of Medicine, Houston, TX USA; 13 Department of Pathology, University of Virginia Health System, Charlottesville, VA USA; 14 Department of Dermatology, University of Virginia Health System, Charlottesville, VA USA; 15 Department of Pathology, Massachusetts General Hospital, Boston, MA USA; 16 Center for Genetic Medicine, Northwestern University, Feinberg School of Medicine, Chicago, IL USA; 17 Robert H. Lurie Comprehensive Cancer Center, Northwestern University Feinberg School of Medicine, Chicago, IL USA

**Keywords:** Gammadelta T cells, Cancer genomics

## Abstract

Primary cutaneous γδ T cell lymphomas (PCGDTLs) represent a heterogeneous group of uncommon but aggressive cancers. Herein, we perform genome-wide DNA, RNA, and T cell receptor (TCR) sequencing on 29 cutaneous γδ lymphomas. We find that PCGDTLs are not uniformly derived from Vδ2 cells. Instead, the cell-of-origin depends on the tissue compartment from which the lymphomas are derived. Lymphomas arising from the outer layer of skin are derived from Vδ1 cells, the predominant γδ cell in the epidermis and dermis. In contrast, panniculitic lymphomas arise from Vδ2 cells, the predominant γδ T cell in the fat. We also show that TCR chain usage is non-random, suggesting common antigens for Vδ1 and Vδ2 lymphomas respectively. In addition, Vδ1 and Vδ2 PCGDTLs harbor similar genomic landscapes with potentially targetable oncogenic mutations in the JAK/STAT, MAPK, MYC, and chromatin modification pathways. Collectively, these findings suggest a paradigm for classifying, staging, and treating these diseases.

## Abstract (web summary)

Cutaneous gamma-delta T cell lymphomas are rare but aggressive cancers. Here, by DNA, RNA and T cell receptor sequencing, the authors elucidate the molecular ontogeny by revising the cell of origin and identifying potentially targetable mutations.

## Introduction

Primary cutaneous γδ T cell lymphomas (PCGDTL) are a heterogeneous group of uncommon but often lethal lymphomas of the γδ T cell^1^. Median survival for patients with PCGDTL is 31 months; 5-year survival is 19.9%^2^. There are no effective therapies for this disease with the lone, possible exception of allogeneic hematopoietic stem cell transplantation^3^.

Despite the near universal agreement about PCGDTL’s poor prognosis, there is no consensus about the disease’s clinical, histological, or molecular features. Patient presentations can be highly variable. Some cases of PCGDTL predominantly involve the epidermis and/or the dermis, while others are centered in subcutaneous adipose tissue^4^. Similarly, the lesions could present as thin patches or thick nodules with or without ulcerations.

The PCGDTL cell of origin, the γδ T cell, represents between 0.5% and 16% of the body’s T cells^5^. Like αβ CD4+ T cells, γδ T cells can express effector cytokines^5^. Like αβ CD8+ T cells, a γδ T cell can also express cytotoxic enzymes, e.g. granzymes and perforin, which cause lysis of both microbes and host cells^6,7^.

Like αβ T cells, γδ T cells undergo VDJ recombination during development; however, unlike for αβ T cells, the choice of Vγ and Vδ segments appears to predict tissue homing and effector function^8^. There are three Vδ segments, but the vast majority of γδ T cells express either Vδ1 or Vδ2 T cell receptors (TCRs). Vδ1 γδ T cells predominate in mucosal interfaces such as the intestinal epithelia. Vδ2 cells represent the majority of circulating γδ T cells in the blood^9–11^. For cutaneous γδ T cell lymphomas (CGDTLs), the cell of origin has long been presumed to be a Vδ2 cell based on Southern Blot analysis of four cases^12^. These findings have not been validated or correlated with clinical phenotype.

Given the aggressive, fatal nature of PCGDTL, there is a critically unmet need to identify putative molecular targets. A single study applied targeted sequencing to 15 cases and identified *STAT3* and *STAT5B* mutations in a minority of samples^13^. Thus, the genetics for this disease remain obscure. To overcome this gap in knowledge, we present a clinical cohort of 42 cases of CGDTLs from four institutions.

To this cohort, we apply DNA sequencing (DNA-Seq) (whole genome [WGS], whole exome [WES], or targeted sequencing) and/or RNA sequencing (RNA-Seq) on 23 cases and TCR sequencing (TCR-Seq) on an additional six cases. Collectively, this analysis identifies 20 putative driver genes including recurrent mutations in the MAPK, MYC, JAK/STAT, and chromatin modification pathways. Our TCR-Seq data suggests that the disease heterogeneity seen in PCGDTL is due in part to distinct cells of origin and effector function status.

## Results

### Clinical presentations

A summary of the cases studied is presented in Supplementary Table 1. Our cases broadly comprise three clinical scenarios. For the first group (25 cases), the diagnosis of PCGDTL was made at the time of clinical presentation. For the second group (16 cases), the patients were originally diagnosed as mycosis fungoides because their clinical and histological features were highly similar to the cutaneous lymphomas of non-cytotoxic αβ T cells. 15/16 of these had patch/plaque stage disease and 1 presented with plaques and tumors. According to the WHO-EORTC criteria, this second group is classified as γδ mycosis fungoides (γδ MF)^1^. A subset of these γδ MF cases (6/16) underwent PCGDTL-like progression. They developed ulcerated, treatment-resistant lesions that were clinically and histologically indistinguishable from PCGDTLs. We define these as γδ MFs with PCGDTL-like progression. The remaining γδ MF cases were identified by TCR-Seq or by immunohistochemistry (IHC) for γδ markers which have become routine at Northwestern. In addition, there was one case of an intravascular γδ T cell lymphoma (IVGDTL) that is presented in the skin (Supplementary Fig. 1). All 42 cases had their γδ TCR lineage confirmed with either IHC and/or TCR-Seq (see “Methods” section). Collectively, we call these CGDTLs.

**Fig. 1:**
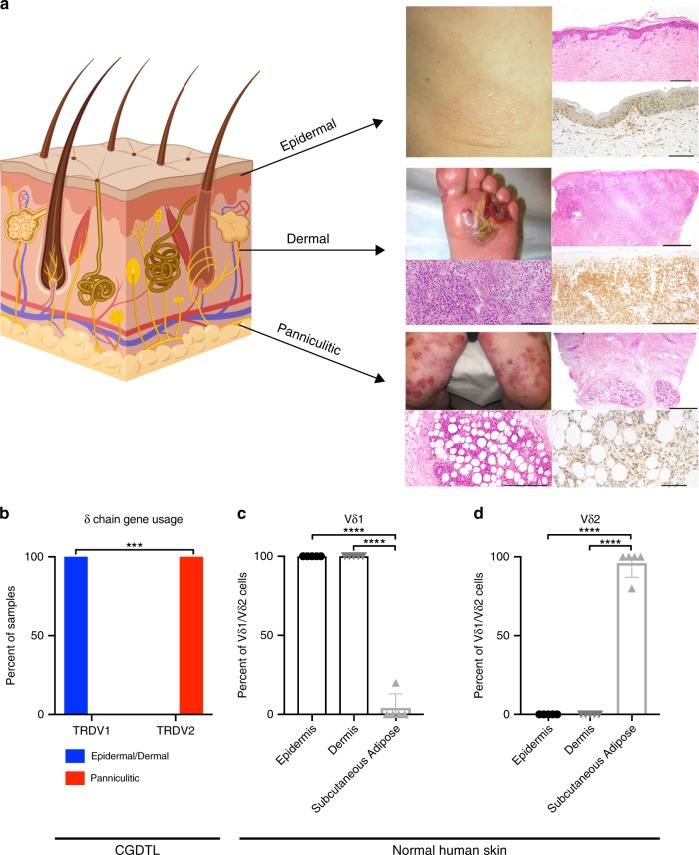
Epidermal/dermal and panniculitic CGDTLs derived from distinct cells of origin. **a** Schematic highlighting distinct clinical and histological presentations of disease involving epidermis, dermis, or subcutaneous tissue. Clinical photographs of disease lesions, hematoxylin and eosin staining of biopsies, and γδ T cell receptor immunostaining (see “Methods” section) for representative patients with epidermal, dermal, and panniculitic disease are shown. Skin schematic created with BioRender. Scale bar represents 100 μm in bottom right epidermal panel, bottom left dermal panel, and bottom right panniculitic panel; 200 μm in top right epidermal panel, bottom right dermal panel, and bottom left panniculitic panel; 500 μm in top right dermal panel and top right panniculitic panel. **b** Frequency of δ chain usage by skin compartment in CGDTL as assessed by RNA-seq and high-throughput TCR-Seq. Lymphomas involving epidermis and/or dermis (*n* = 8) or subcutaneous tissue (panniculitic) (*n* = 7). *** Indicates *P* value = 0.0002, two-sided Fisher’s exact test. **c**, **d** Flow cytometry analysis showing percentage of Vδ1 and Vδ2 T cells in normal human epidermis, dermis, and subcutaneous tissue (*n* = 5). Dots represent individual values, horizontal line represents mean, and error bars represent standard deviation. **** Indicates *P* value < 0.0001, one-way ANOVA followed by Tukey’s multiple comparison test. Source data are provided as a source data file.

The clinical–histological presentations were heterogeneous. The lesions manifested clinically as ulcerated or non-ulcerated patches, plaques, or nodules. On pathological examination, the tumor infiltrates involved the epidermis, dermis, and/or subcutaneous tissue. A schematic of the depth of predominant tumor involvement and corresponding clinical photographs, hematoxylin and eosin staining, and γδ TCR immunostaining are presented in Fig. 1a. The tumor cells were CD3+ but negative for markers of αβ T cells with few exceptions (Supplementary Table 2). Other markers were variably expressed. For example, there was wide variability in the expression of cytotoxic markers. 33 of the 42 cases had available IHC for cytotoxic markers (TIA-1, granzyme B, perforin). Of these, 79% (26/33) cases expressed at least one cytotoxic marker whereas 21% (7/33) tested negative. Biopsies from two subjects were initially negative but eventually acquired expression of cytotoxic markers in a subsequent tissue sample.

### Next generation sequencing identifies distinct cells of origin

From these cases, we identified 29 samples with sufficient material for molecular analyses (Supplementary Table 3). These include 16 PCGDTLs, 2 γδ MFs that retained their original non-cytotoxic clinical phenotypes, 4 γδ MFs with PCGDTL-like progression, and 1 IVGDTL. 12 samples were sent for DNA-Seq (WGS, WES, or targeted sequencing) alone, 3 samples for RNA-Seq alone, and 8 samples for both DNA-Seq and RNA-Seq. DNA and RNA were isolated from needle cores of tumor-rich regions from formalin-fixed paraffin-embedded blocks or from fresh frozen tumor biopsies (see section “Methods”, Supplementary Table 3). Two samples subject to targeted sequencing also underwent TCR-Seq (see below). Whole tissue sections or fresh frozen biopsy from six samples were subject to TCR-Seq alone.

Because the clinicopathological presentations were variable, we first confirmed the diagnosis using TCR-Seq. For samples with RNA-Seq and WGS data, we utilized a previously published algorithm, MiXCR^14^, to infer TCR sequences (Supplementary Figs. 2, 3, Supplementary Table 4). MiXCR was successfully run on 9 of 11 samples with RNA-Seq. In all nine of these cases, the top hit was a γδ TCR gene. To confirm these results, we performed orthogonal analyses. MiXCR was successfully run on WGS data for 3 of 6 samples (Supplementary Table 4). These data confirmed the same γδ TCR clonotypes identified by RNA-seq. In addition, for one of these three samples, we confirmed the MiXCR results with additional experiments, namely flow cytometry and γδ TCR gene single-cell RNA sequencing (scRNA-Seq) (see “Methods” section, Supplementary Fig. 2).

Based on the literature^1,12^, we had anticipated that all cases would be of Vδ2 origin. Indeed, this was true for 4 of our 9 cases. Surprisingly, we found that the predominant γδ TCR in the remaining 5 samples were Vδ1. To confirm these results, we performed high-throughput TCR-Seq (see “Methods” section) on the genomic DNA from 8 additional samples. For 6 of these samples, δ chain usage was successfully determined (Supplementary Fig. 4, Supplementary Table 6). Collectively, we found that 53% (8/15) were Vδ1 and 47% (7/15) were Vδ2. The Vδ chain for the intravascular case could not be resolved.

The cell of origin was non-random. Strikingly, the center of gravity in 100% (8/8) of the Vδ1 lymphomas was either in the epidermis or the dermis (Fig. 1b). These include γδ MFs, γδ MFs with PCGDTL-like progression, and PCGDTLs. In contrast, all of the Vδ2 lymphomas (7/7) were primarily in the subcutaneous tissue (panniculitic PCGDTL). The association between cell of origin and depth of pathological infiltrate was statistically significant (*P* = 0.0002; Fisher’s exact test). On the basis of these data, we henceforth refer to lymphomas originating in the epidermis/dermis and fat as Vδ1 and Vδ2 lymphomas, respectively.

We hypothesized that the cell of origin reflected the predominant cell type in each compartment in non-diseased skin. Because of disagreements in the literature about the predominant γδ T cell in normal human skin^15–17^, we performed flow cytometric analysis on single cell suspensions made from human skin from five healthy donors (Supplementary Fig. 5). Consistent with our findings in γδ lymphomas, both epidermis and dermis were enriched for Vδ1 cells (*P* < 0.0001; one way ANOVA followed by Tukey’s multiple comparisons test) (Fig. 1c), while subcutaneous fat was enriched for Vδ2 cells (*P* < 0.0001; one way ANOVA followed by Tukey’s multiple comparisons test) (Fig. 1d).

**Fig. 2:**
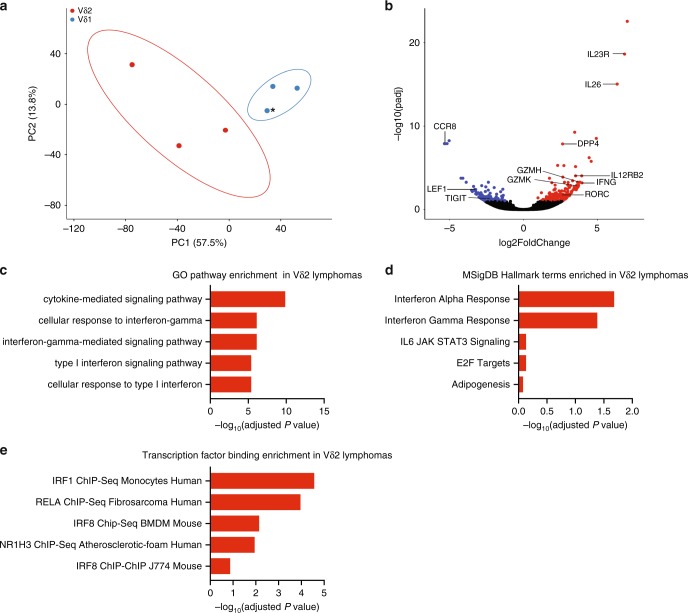
Vδ1 and Vδ2 CGDTL lymphomas are transcriptionally distinct. **a** Principal component analysis of transcriptomes of Vδ1 and Vδ2 PCGDTL samples. * Indicates Vδ1 γδ MF with PCGDTL-like progression sample. **b** Volcano plot of differentially expressed genes significantly upregulated in Vδ1 (blue) and Vδ2 (red) with an adjusted *P* value < 0.05 (DESeq2). Select immune-related genes are labeled. **c**–**e** Top five pathways upregulated in Vδ2 PCGDTL lymphomas via GO pathway, MSigDB Hallmark, and CheA transcription factor-binding analysis respectively.

To determine the impact of cell of origin on tumor phenotype, we analyzed the RNA-Seq data for six samples processed similarly and in one batch (3 Vδ1 epidermal/dermal lymphomas vs. 3 Vδ2 panniculitic lymphomas) (see “Methods” section). By principal component analysis, they clustered separately, suggesting they are transcriptionally distinct (Fig. 2a). Notably, the γδ MF with PCGDTL-like progression clustered with the two Vδ1 PCGDTLs. Differential gene expression analysis suggested that there were 61 and 138 genes enriched in the Vδ1 and Vδ2 lymphomas, respectively (adjusted *P* value < 0.05; DESeq2^18^) (Fig. 2b, Supplementary Data 1).

To assess the effect of cell of origin on transcriptomes, we compared our results with a recent RNA-Seq dataset comparing circulating Vδ1 and Vδ2 γδ T cells in the blood^19^. As expected, the Vδ2 lymphomas in our cohort had significantly higher expression of genes encoding Vδ2-specific genes. These include cell-surface markers (*IL12RB2*, *IL23R*, and *CD26*) and the transcription factor *RORC*. In contrast, the Vδ1 lymphomas had elevated expression of transcripts that were also elevated in untransformed, circulating Vδ1 cells, including the transcription factor *LEF1* and the cell-surface receptor *TIGIT*. Consistent with their localization to the epidermis and dermis, Vδ1 lymphomas expressed significantly increased levels of *CCR8* compared to Vδ2 lymphomas, a chemokine receptor normally found on untransformed epidermis and dermis-resident memory T cells^20^.

The RNA-Seq analysis also showed an enrichment of cytotoxic and inflammatory genes in the Vδ2 lymphomas. These include granzymes (such as *GZMK* and *GZMH*), Th1-associated genes (*IFNG* and *STAT4*), and Th17-associated genes (*IL26*, *IL23R*, and *RORC*). Pathway analysis [gene ontology (GO) analysis^21^ and gene set variation analysis (GSVA)^22^] confirmed that the Vδ2 lymphomas had significantly higher expression of pathways involving type I interferon or interferon-γ (IFN-γ) (adjusted *P* value = 4 × 10^−6^, 0.04, respectively) (Fig. 2c, d, Supplementary Tables 7 and 8). ChEA analysis^23^ confirmed that Vδ2 genes had significant enrichment of genes downstream of interferon-associated transcription factors, e.g. IRF1 (adjusted *P* value = 3 × 10^−5^, 7 × 10^−3^ respectively) (Fig. 2e, Supplementary Table 9).

### Cell of origin and histology affects clinical phenotype

We hypothesized that the differences in the cell of origin contributed to the heterogeneity of clinical presentations. We therefore examined the clinical characteristics of these two cohorts. The median age, ethnicity, and gender of the patients were similar between these two cohorts (Supplementary Table 1). Similarly, both tumor types favored the same anatomical locations, i.e. the legs over the trunk and arms (Supplementary Table 1).

**Fig. 3:**
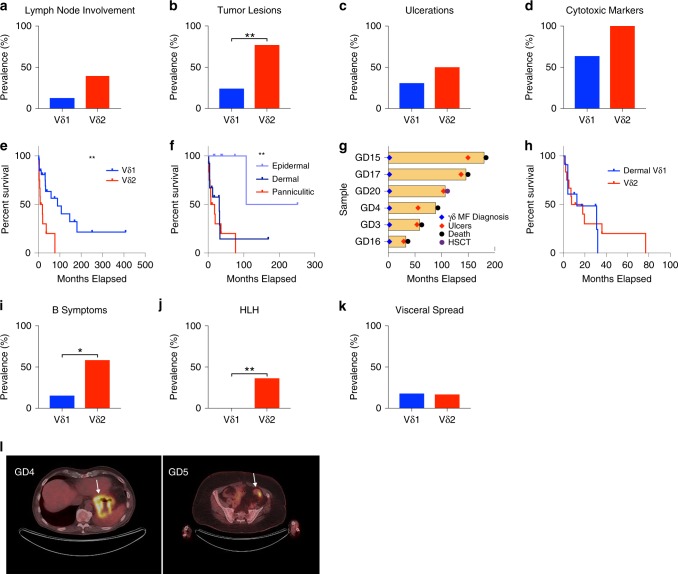
Cell of origin and histologic subtype influences clinical phenotypes in CGDTL. **a**, **b** Prevalence in patients with Vδ1 or Vδ2 disease of lymph node involvement (Vδ1 *n* = 24; Vδ2 *n* = 10) and tumor stage skin lesions (Vδ1 *n* = 25; Vδ2 *n* = 13) at diagnosis, respectively. **c**, **d** Prevalence of ulcerated skin lesions (Vδ1 *n* = 26; Vδ2 *n* = 12) and cytotoxic marker staining (Vδ1 *n* = 22; Vδ2 *n* = 9), respectively, based on cell of origin. **e** Kaplan–Meier curves of patients with Vδ1 (*n* = 27) or Vδ2 (*n* = 12) lymphomas. **f** Kaplan–Meier curve based on depth of disease in the diagnostic biopsy specimen (epidermal *n* = 9; dermal *n* = 13; panniculitic *n* = 12). **g** Swimmer’s plot highlighting disease course of Mycosis-fungoides like cases that acquire an ulcerative phenotype (*n* = 6). HSCT, hematopoietic stem cell transplant. **h** Kaplan–Meier survival curve for patients with dermal Vδ1 lymphomas (*n* = 11; excluding γδ MF cases) and Vδ2 lymphomas (*n* = 12). **i**–**k** Frequency of B symptoms (Vδ1 *n* = 26; Vδ2 *n* = 12), hemophagocytic lymphohistiocytosis (HLH) (Vδ1 *n* = 25; Vδ2 *n* = 11), and visceral spread (Vδ1 *n* = 28, Vδ2 *n* = 12), respectively, among patients with Vδ1 or Vδ2 disease. **l** Images of positron emission tomography–computed tomography from two patients with Vδ1 lymphomas with spread to the gastrointestinal tract. White arrows highlight areas of increased signal; stomach (GD4) and large intestine (GD5). For e, f ** indicates *P* value <0.005, log-rank test. For **b**, **i**, **j** *indicates *P* value <0.05, and **indicates *P* value <0.005, two-sided Fisher’s exact test. Source data are provided as a source data file.

Adverse prognostic factors in other cutaneous lymphomas^24^ were more common in the Vδ2 lymphomas, e.g. lymph node involvement (40% vs. 13%) (*P* = 0.0767; Fisher’s exact test), although these measures did not all reach statistical significance (Fig. 3a, b). Furthermore, consistent with the RNA-Seq data, the Vδ2 lymphomas had a higher rate of clinical or molecular features of cytotoxicity (Fig. 3c, d).

We then examined the effect of cell of origin on disease prognosis (Fig. 3e). Expanding on prior literature^4^, we found that median survival for the Vδ1 and Vδ2 lymphomas is 89 and 12.75 months, respectively (*P* = 0.002; log-rank test).

Upon closer inspection, there were two factors that contributed to the cell-of-origin dependent differences in survival: the depth of disease and clinical phenotype. The median survival was 179 months for lesions centered on the epidermis (Vδ1), which is significantly longer than that for lesions in the dermis (Vδ1) and subcutaneous fat (Vδ2) at 31 and 12.75 months, respectively (*P* = 0.007; *P* = 0.0006; log-rank test) (Fig. 3f).

The γδ MF phenotype, which was exclusively found in the Vδ1 lymphomas, was associated with a better prognosis. All γδ MFs initially responded to skin-directed therapies or conservative biologics, such as interferon-α or retinoids, with some cases achieving a complete remission. 63% (10/16) of the γδ MFs retained their non-ulcerated, indolent phenotype throughout their disease course. None of these patients died due to disease (median follow-up: 38 months).

However, the γδ MF disease course is dynamic. 37% (6/16) progressed after a prolonged indolent phase (median follow-up: 98 months) (Fig. 3g). Once these cases progress, the clinical phenotype and overall prognosis becomes indistinguishable from those newly diagnosed with PCGDTL. These cases no longer resemble MF, a disease typically associated with the non-cytotoxic CD4+ T cell. Instead all of these patients developed ulcerations, a clinical hallmark of cytotoxic lymphomas such as PCGDTLs^25^. The disease becomes recalcitrant to systemic therapies including systemic chemotherapy and immunomodulatory therapies (Supplementary Table 10). 5 of these 6 patients (83%) had a fatal disease course. The sixth received an allogeneic bone marrow transplant for progressive disease.

Excluding the γδ MFs, there were no other epidermal Vδ1 lymphomas. Now, the prognosis in these dermal Vδ1 PCGDTLs were no longer different from panniculitic Vδ2 PCGDTLs (median survival = 12.5 vs. 12.75 months) (*P* = 0.85; log-rank test) (Fig. 3h). Moreover, median survival for γδ MFs with PCGDTL-like progression was 16.5 months after the phenotypic switch, which is in line with that of newly diagnosed Vδ1 PCGDTLs (12.5 months). Collectively, these data suggest that the presence of a PCGDTL-like phenotype, which is uniform in Vδ2 lymphomas and sporadic in Vδ1 lymphomas, drives cell-of-origin-dependent differences in survival.

There were other cell-of-origin-dependent disease features. Consistent with the increased expression of cytokines observed by RNA-Seq (Fig. 2b), the Vδ2 lymphomas had a higher incidence of cytokine-driven syndromes. There was a significantly greater prevalence of B symptoms (fevers, night sweats, and/or weight loss) at diagnosis (58% vs. 15%, respectively) (*P* = 0.02; Fisher’s exact test) (Fig. 3i). In addition, a subset of patients with PCGDTLs eventually developed hemophagocytic lymphohistiocytosis (HLH), a potentially fatal inflammatory syndrome characterized by overproduction of inflammatory cytokines, such as tumor necrosis factor α (TNF-α), IFN-γ, interleukin-1 (IL-1), and interleukin-6 (IL-6)^25,26^. HLH did not occur in Vδ1 lymphomas (36% vs. 0%) (*P* = 0.005; Fisher’s exact test) (Fig. 3j).

Lastly, a subset of PCGDTL patients developed visceral metastases (Supplementary Table 2). Visceral organ involvement was similar in both lymphoma types (18% vs. 16% in the Vδ1 and Vδ2 lymphomas, respectively) (*P* = 0.7; Fisher’s exact test) (Fig. 3k). Metastatic disease was observed in both patients with PCGDTL and γδ MF with PCGDTL-like progression. The most common sites in both Vδ1 and Vδ2 lymphomas were the liver or spleen (12% of all CGDTLs), intestine (7%) and central nervous system (7%) (Supplementary Table 1). Notably, metastases to the gut epithelium, home to epithelial homing Vδ1 γδ T cell^27^, were exclusively found in lymphomas of the Vδ1 γδ T cell (Fig. 3l, Supplementary Table 11).

### Putative antigen specificity of CGDTL

**Fig. 4:**
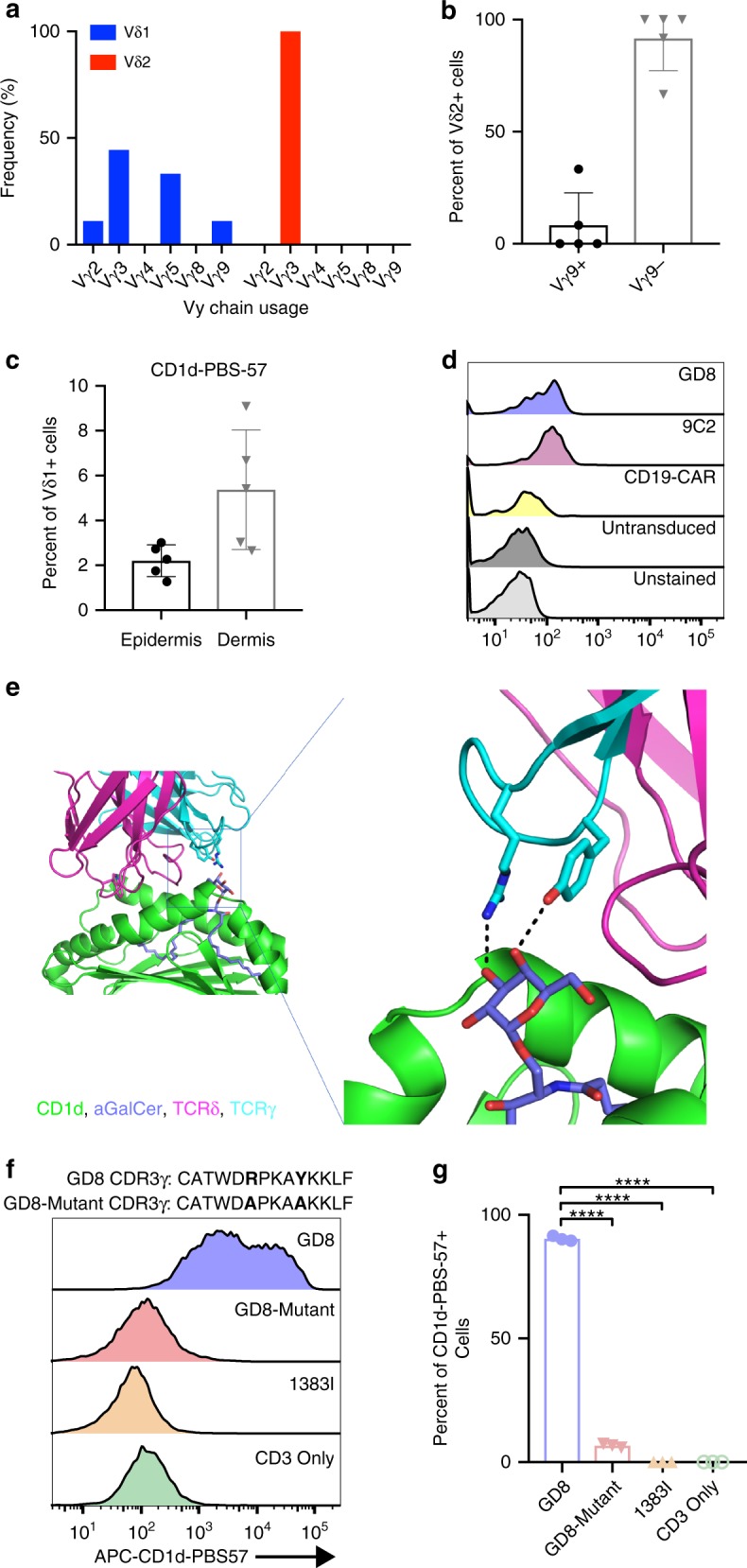
CD1d-lipid binding of Vδ1 CGDTL TCR. **a** Frequency of γ chain usage among PCGDTL and γδ MF by disease subtype. **b** Frequency of Vγ9+ and Vγ9− cells among Vδ2 cells from subcutaneous adipose tissue in normal donors (*n* = 5). **c** Frequency of CD1d-PBS-57 tetramer staining, gated on Vδ1 cells. *n* = 5 donors in each condition. **d** Flow cytometry histogram of retrovirally transduced CD8+ T cells with fluorescently labeled CD1d-PBS-57 tetramers. **e** Structural model of GD8 TCR in complex with CD1d-lipid. On right, CDR3γ residues Arg 103 and Tyr 107 are shown as sticks, with predicted polar interactions with glycolipid head group shown as dotted lines. **f** Representative histogram of flow cytometry analysis of CD1d-PBS-57 tetramer staining in HEK293 cells co-transfected with CD3 and TCR. Amino acid sequence of GD8 and GD8-Mutant CDR3γ are indicated. **g** Percent of CD1d-PBS-57 tetramer positive cells in HEK293 cells co-transfected with TCR and CD3. *n* = 3 independent experiments, **** indicates *P* value <0.0001, one way ANOVA followed by Tukey’s multiple comparisons test. Dots represent individual values, horizontal line represents mean, and error bars represent standard deviation. Source data are provided as a source data file.

Further analysis of the TCR-Seq data suggested that the Vγ chain usage within each group was also non-random (Fig. 4a). There are six functional Vγ chains in the TCR gamma locus, and the vast majority of circulating γδ T cells express Vγ9^8^. All Vδ2 PCGDTLs in our cohort (6/6) expressed Vγ3 and not Vγ9. Similarly, Vδ2 cells from normal human subcutaneous tissue were largely negative for Vγ9, the only γ chain for which an antibody is available (Fig. 4b). Of Vδ1 samples with γ chain usage resolved, 78% (7/9) expressed Vγ3 (*n* = 4) or Vγ5 (*n* = 3); Vγ2 or Vγ9 were expressed in one case each.

We hypothesized that biased usage of γ and δ TCR variable chains suggests a common antigen for the Vδ1 and Vδ2 lymphomas, respectively. To explore this further, we first compared our TCR complementary determining region (CDR3) sequences with published CDR3s of γδ T cells with known antigen specificity. We observed similarities between Vδ1 PCGDTL CDR3γ sequences and Vδ1 cell CDR3γ sequences from normal human peripheral blood which bind to CD1d–α-galactosylceramide^28^ (Supplementary Table 12). CD1d is a major histocompatibility-like molecule which can present lipid antigens to γδ T cells and is expressed by dermal dendritic cells in normal human skin^29^. Notably, flow cytometry using CD1d tetramers loaded with PBS-57 (an α-galactosylceramide analog), showed a subset of Vδ1 cells bind to CD1d-PBS-57 in normal human epidermis and dermis (Fig. 4c). Therefore, we hypothesized that at least a subset of the Vδ1 PCGDTLs affecting the epidermis and dermis may be CD1d-restricted.

To test whether the γδ TCR in PCGDTL patients could bind to CD1d in complex with lipid antigen, we retrovirally transduced normal CD8+ T cells with the Vγ5/Vδ1 TCR from a patient with epidermal/dermal disease (GD8). As a positive control we transduced the 9C2 TCR, a Vγ5/Vδ1 TCR previously shown to bind to CD1d-α-galactosylceramide by binding assays and by crystal structure^28^. Indeed, both 9C2 and GD8 TCR-expressing cells bound to CD1d-PBS-57 tetramers in vitro, while cells expressing a chimeric antigen receptor recognizing CD19 (CD19-CAR) or un-transduced cells did not (Fig. 4d).

To probe molecular mechanisms, we modeled GD8 TCR-binding CD1d in complex with lipid antigen, utilizing the similar, previously published 9C2 crystal structure (Protein Data Bank: 4LHU). Interestingly, we observed that tyrosine and arginine residues critical for hydrogen bonding with the polar head group of the lipid antigen in the CDR3γ of the 9C2 TCR were conserved in GD8 and could form hydrogen bonds with the antigen in our model (Fig. 4e). Nonetheless, the presence of this arginine/tyrosine motif does not appear to be a requirement for CD1d-lipid antigen recognition by all γδ TCRs as it does not occur in 57% of CD1d-lipid-binding γδ TCRs^28^.

Consistent with this model, 33% of Vδ1 tumors had CDR3s with arginine and tyrosine at the antigen-binding interface (Supplementary Table 12). To test whether these motifs were required for CD1d-lipid antigen binding, we mutated these amino acids to alanine (GD8-Mutant). For higher expression^30^ we transduced HEK293 cells with retroviral vectors encoding GD8, GD8-Mutant, or the melanoma-specific αβ TCR 1383I utilizing a recently published protocol (see “Methods” section). Surface expression of the TCRs were roughly equivalent across all cell lines (Supplementary Fig. 6). As expected, the tetramer failed to bind the negative controls, i.e. the cells expressing the αβ TCR or no TCR. Consistent with our prior experiments, the tetramer bound to the HEK293 T cells expressing the GD8 TCR. However, this binding was abrogated by the arginine/tyrosine to alanine mutations in GD8-Mutant (*P* < 0.0001, one-way ANOVA followed by Tukey’s multiple comparisons test) (Fig. 4f, g).

### Frequent mutations in MAPK, MYC, JAK/STAT, and chromatin-remodeling pathways in CGDTLs

**Fig. 5:**
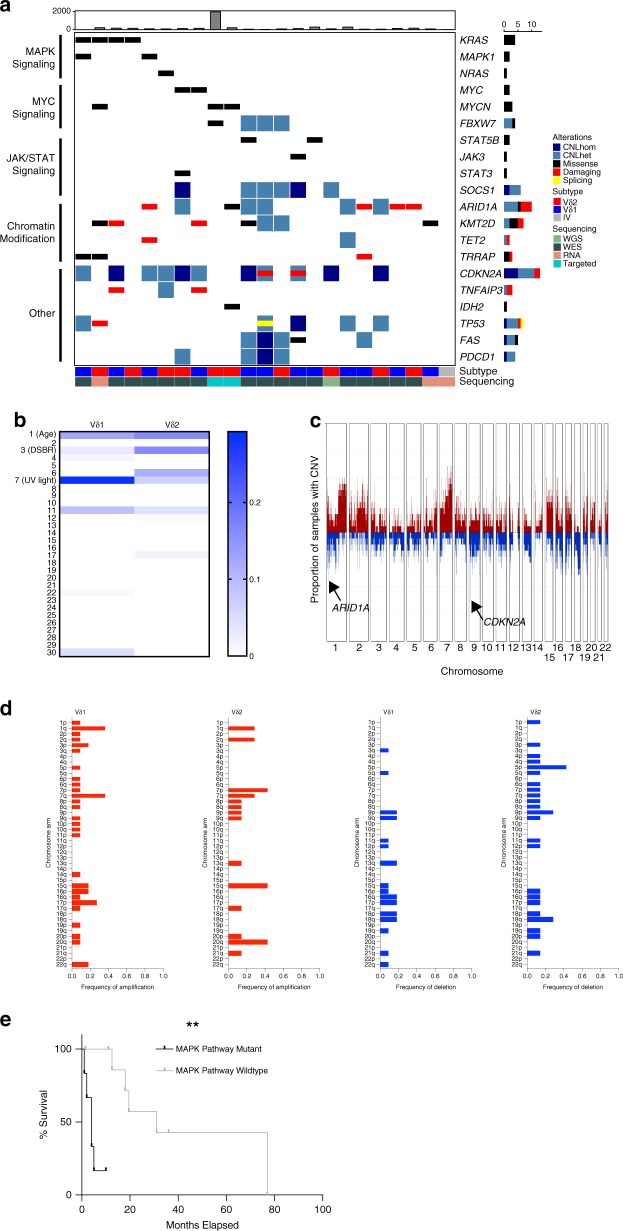
The genomic landscape of CGDTL. **a** Putative driver mutations in CGDTL. Rows represent genes, and each column corresponds to a CGDTL sample. Top bar plot indicates number of non-synonymous mutations detected in each sample, and bar plot on the right indicates total number of mutations in each putative driver gene. CNLhom and CNLhet; homozygous and heterozygous copy number loss, respectively. **b** Median contribution of COSMIC mutational signatures in CGDTL subtypes, Vδ1 *n* = 11, Vδ2 *n* = 7. DSBR double-strand break repair defect. **c** Frequency of copy number alterations. Driver genes within significant GISTIC peaks are listed. **d** Frequencies of arm level events in CGDTL subtypes. **e** Kaplan–Meier curve depicting percent survival with (*n* = 6) or without (*n* = 9) recurrent hotspot mutations in MAPK pathway genes (*KRAS*, *MAPK1*, and *NRAS*). ** Indicates *P* value < 0.005, log-rank test.

The ontogeny of lymphomas involves both cellular context and somatic mutations. We therefore next analyzed WES, WGS, targeted sequencing, and/or RNA-sequencing data from 23 patients (see “Methods” section, Fig. 5a). Quality control suggested a median tumor purity of 88% (see “Methods” section, Supplementary Table 13). Each sample harbored a median of 138 non-synonymous somatic single nucleotide variants (SSNVs) (Supplementary Table 14, Supplementary Data 2). CGDTL genomes were highly unstable, as we detected a median of 166.5 somatic copy number variants (SCNVs) per sample, including a median of four arm level events per sample (Supplementary Table 13, Supplementary Data 3). Interrogation of the RNA-Seq data with PRADA software^31^ failed to identify bona-fide gene fusions.

We then examined the SSNVs for mutational signatures (see “Methods” section, Fig. 5b, Supplementary Fig. 7). Signature 7, which is associated with exposure to UV light, was the most prevalent signature identified in Vδ1 lymphomas, which are centered in the upper layers of the skin. In contrast, Vδ2 samples, which are derived from the presumably sun-shielded subcutaneous fat, were most enriched for signature 1 (associated with aging), followed by signature 3 (associated with defects in DNA double strand break repair).

Collectively, we identified 20 putative driver genes (Fig. 5a). These results confirmed the presence of *STAT3* and *STAT5B* mutations^13^ and identified an 18 additional putative drivers in CGDTLs. These mutations affect multiple, oncogenic pathways, including MAPK signaling (*KRAS*, *NRAS*, *MAPK1*), MYC pathway (*MYC*, *MYCN*, *FBXW7*), JAK/STAT signaling (*STAT3*, *STAT5B*, *JAK3*, *SOCS1*), and chromatin modification (*ARID1A*, *TRRAP*, *TET2*, *KMT2D*). Additional mutations affect consensus cancer genes (*CDKN2A*, *IDH2*, *TP53*) as well as tumor suppressors previously identified in cutaneous T cell lymphoma (*TNFAIP3*, *FAS*, *PDCD1*). Alterations in at least one driver gene were detected in each sample with the lone exception of a case with intravascular disease analyzed by RNA-sequencing.

*KRAS* was the most frequently mutated putative oncogene. Mutations in *KRAS* occur predominantly in previously functionally validated hotspots (p.G12D, p.Q61H, and p.D119N)^32,33^. We identified recurrent mutations both in *MYC* (p.P74L, previously reported as recurrent in acute myeloid leukemia patients^34^) and *MYCN* (p.G34R). Lastly, consistent with previous reports of PCGDTLs^13^, we observed point mutations in JAK/STAT signaling in 21% of samples. These include the JAK/STAT mutations previously reported in T cell cancers (*STAT3* p.D661V, *STAT5B* p.P702A, and *JAK3* p.R657W)^35–37^. Schematics of select mutations in putative oncogenes are presented in Supplementary Fig. 8.

To examine whether noncoding mutations may play a role in CGDTLs, we examined the DNA-Seq data for validated non-coding mutations. We found that 14% of samples with sufficient coverage harbored known gain-of-function mutations (Supplementary Table 15). The *TERT* gene encodes the protein telomerase, which maintains telomere length. These mutations lead to higher expression of hTERT^38^.

Next, we analyzed copy number information for 18 CGDTLs with interpretable copy number data (Fig. 5c, Supplementary Fig. 9). Analysis of SCNVs in CGDTL revealed recurrent arm level amplifications and deletions, including amplification of 1q (33%), 15q (33%), and 7q (39%) and deletions of 9p (22%) and 18q (22%) (Supplementary Data 3). To identify putative tumor suppressors and oncogenes residing on SCNVs, we utilized GISTIC2 (Supplementary Table 16)^39^. GISTIC analysis identified significantly recurrent deletions of *CDKN2A* (deleted in 61% of samples, with 45% of deletions biallelic) and *ARID1A* (deleted in 28% of samples). Significantly recurrent amplifications included *TNFRSF1B* (amplified in 33%), which encodes the NF-kB pathway activating oncogene *TNFR2*^40^, and *MAP4K4* (amplified in 17%), an activator of ERK signaling associated with poor outcomes in numerous cancers^41^. In addition, we found focal deletions common in other T cell cancers^42,43^ (*FAS* and *PDCD1*) in 22% of cases each, one sample each with biallelic deletion of *FAS* or *PDCD1*.

Based on our clinical and transcriptional data, we hypothesized that genetic drivers may be different between the Vδ1 and Vδ2 derived lymphomas. Unexpectedly, we did not observe statistically significant genetic differences between the groups. Point mutations in putative driver pathways, such as *MAPK*, *JAK-STAT*, and *MYC* were not exclusive to one group, as both Vδ1 and Vδ2 cases had alterations in each pathway (Fig. 5a). Copy number mutations also were similar between the two subgroups (Fig. 5d).

**Fig. 6:**
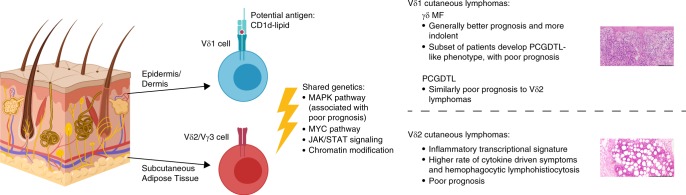
Proposed model of CGDTL cell of origin and disease pathogenesis. Epidermal/dermal disease arises from the Vδ1 cell and can potentially bind to lipid antigens presented by CD1d. Panniculitic disease arises from the Vδ2/Vγ3 cell in the subcutaneous tissue. Both cells acquire similar genetic alterations. Vδ1 γδ MFs clinically can have either a non-cytotoxic, indolent course or can switch after prolonged indolence to a cytotoxic phenotype that is more aggressive. Vδ2 lymphomas are more aggressive, associated with inflammatory gene signatures and the development of cytokine-driven paraneoplastic syndromes including hemophagocytic lymphohistiocytosis. Scale bar represents 100 μm (top) and 200 μm (bottom). Created with BioRender.

We performed similar analyses between the Vδ1 γδ MFs with PCGDTL-like progression (*n* = 4) and the Vδ1 PCGDTLs (*n* = 6). There were differences in MAPK pathway (*KRAS*, *NRAS*, *MAPK1*) mutations, however none of these reached statistical significance. These mutations are defined in Supplementary Tables 17 and 18. Collectively, our data suggests a model wherein the CGDTLs develop via the acquisition of similar genetic mutations from two distinct cells of origin (Fig. 6).

Next, we aimed to assess the clinical implications of our genetic findings (Supplementary Data 4). We looked for genetic biomarkers previously implicated in cutaneous lymphomas^44,45^. We did not observe significant differences in overall survival among *CDKN2A* deleted compared to wild-type PCGDTLs (*P* = 0.43, log rank test). Interestingly, MAPK pathway mutations, i.e. gain-of-function SSNVs in *KRAS*, *NRAS*, and *MAPK1*, were associated with worse overall survival in CGDTLs (*P* = 0.001, log rank test) (Fig. 5e).

We then cross-referenced our data with precision medicine databases to identify clinically actionable mutations^46^. 24% of our WES cohort harbored potentially targetable mutations in MAPK signaling and JAK/STAT signaling (Table 1). These mutations have conferred sensitivity to MEK, combined MEK/AKT, or JAK inhibition in preclinical models and clinical trials^37,47,48^.

**Table 1:** Putative clinically actionable mutations detected in CGDTL.

Gene	Mutation	Evidence	Disease	Targetability	Pubmed ID
*KRAS*	p.G12D	Clinical trial	Advanced solid stage tumors	MK-2206 and selumetinib (AKT/MEK co-inhibition)	25516890
*NRAS*	p.Q61H	Clinical trial	Advanced melanoma	Binimetinib (MEK inhibitor)	28284557
*MAPK1*	p.E322K	Case report	Head and neck squamous cell carcinoma	Erlotinib (EGFR inhibitor)	27004400
*JAK3*	p.R657W^a^	Preclinical model	Acute myeloid leukemia	Tofacitinib or ruxolitinib (JAK inhibitors)	25193870

^a^Indicates mutation involving same amino acid position, but not same amino acid change, tested.

### Genetic similarities and differences between PCGDTL, γδ MF, and other T cell lymphomas

We compared the distribution of mutations in γδ MFs without PCGDTL-like progression, γδ MFs with PCGDTL-like progression, and PCGDTL with other cutaneous lymphomas^49^ and other γδ T cell cancers^36,50^. There were broad similarities with these other lymphomas, e.g. activating mutations in the JAK/STAT pathway.

Of note, we failed to identify germline or somatic mutations in genes associated with genetic HLH syndromes. These include *HAVCR2*, which encodes the co-inhibitory receptor TIM-3, and is a cause of HLH among a majority of patients with αβ SPTLs^49^. In addition, we did not detect damaging mutations or copy number losses in *SETD2*, the most frequently silenced gene in both hepatosplenic T cell lymphoma (HSTCL) and enteropathy-associated T cell lymphoma type II^36,50^.

Lastly, despite the similar diagnosis in WHO-EORTC criteria, we failed to identify mutations in the TCR-CD28 pathway found in αβ MFs, such as *CD28*, *PLCG1*, *VAV1*, and *CARD11*^40^.

## Discussion

Collectively, our studies have elucidated the cell of origin and the genetics of this uncommon but aggressive disease. In contrast to current dogma, we found that γδ T cell lymphomas arising from different skin compartments have distinct cells of origin. Broadly, these lymphomas have the molecular features of their skin-resident cells of origin though these comparisons are limited due to insufficient exposition of the features of skin-resident γδ T cells. For example, the RNA-Seq data has been restricted to-date to circulating Vδ1 and Vδ2 cells^19^.

Differences in cell of origin appear to contribute to the heterogeneity of the clinical and pathological phenotypes. The Vδ1 γδ T cell is the cell of origin for the epidermal/dermal γδ T cell lymphomas. Consistent with their epithelial-homing cell of origin, Vδ1 CGDTLs have a UV mutational signature. Moreover, they uniquely have the potential to metastasize to other Vδ1 γδ T cell homing sites, such as the gut mucosa. Lastly, like other Vδ1 cancers, such as HSTCL, there is variable expression of cytotoxic markers^1,51^.

The Vδ2 γδ T cell is the cell of origin for the panniculitic γδ T cell lymphomas. Consistent with a subcutaneous cell of origin, these tumors do not express a UV mutational signature. These lymphomas uniformly express cytotoxic markers, are more aggressive, and have a significantly increased expression of IFN-γ and other inflammatory cytokines. We hypothesize that the significant enrichment of inflammatory cytokines in the Vδ2 cases contributes to the increased risk of cytokine-driven symptoms. In particular, the significant upregulation of IFN-γ in Vδ2 lymphomas is consistent with emerging data suggesting a causal role for the cytokine in HLH and the potential therapeutic utility of IFN-γ blocking antibodies^52^.

Because of our sample size (*n* = 15), we cannot rule out Vγ chain usage not observed in our study. Nonetheless, the γ chains also appear to be strikingly non-random. All the Vδ2 lymphomas in our cohort had an accompanying Vγ3 chain, which is an uncommon finding in the peripheral blood^53,54^. The vast majority (7/9; 78%) of the Vδ1 lymphomas had an accompanying Vγ3 or Vγ5 chain. We speculate that these data suggest a common antigenic trigger for each tumor subtype. In particular, our data suggests that for Vδ1 lymphomas the antigen may be a lipid presented by CD1d. The target antigen for the Vγ3Vδ2 TCR of panniculitic PCGDTLs remains obscure but may be a soluble antigen such as histidyl-tRNA synthetase^55^. Another possibility is the existence of a yet-undiscovered restriction factor in the fat for the Vγ3 chain, similar to that for Vγ4 intra-epithelial lymphocytes in the intestine^56^.

The shared TCR chains suggests the importance of chronic TCR stimulation for this disease. Consistent with this hypothesis, four CGDTLs had deletions involving *PDCD1*, a gene which encodes an inhibitory receptor upregulated after chronic TCR signaling^57^. If true, these data have important therapeutic applications. In mouse models, for example, CD1d–TCR interactions can be targeted with blocking antibodies^58^.

Moreover, our findings suggest the potential need to revisit disease classifications. Current WHO-EORTC criteria subclassifies skin-based γδ T cell lymphomas into γδ mycosis fungoides and PCGDTL based on the clinical and histological findings at diagnosis. Our study suggests that these two clinical entities may be linked. As an example, 38% of γδ MFs in our cohort develop lesions that are clinically, genetically, and transcriptionally indistinguishable from PCGDTLs. Because we cannot predict which γδ MF cases will acquire the aggressive, PCGDTL-like clinical phenotype, we suggest that all CGDTLs be classified together (Supplementary Table 19). The spectrum of disease, i.e. the contribution of ulcerations/cytotoxic phenotypes and depth of infiltrates, can be clinically accounted for in the staging criteria.

Understandably, changing WHO-EORTC criteria requires careful consideration especially because current WHO-EORTC lymphoma criteria do not group diseases together based on cell of origin alone. Additionally, prospective multi-center studies may be needed to validate our findings and to overcome possible sources of biases related to the retrospective, oligo-institutional design of our study. Specifically, the lack of routine γδ TCR marker testing at the time of MF diagnosis creates a possible selection bias. This bias enriches for γδ MF cases with PCGDTL-like progression and may exclude γδ MFs without progression.

In this study, we elucidate the landscape of genetic alterations which drive this aggressive disease. This genomic map can be leveraged for precision medicine. We have uncovered therapeutically actionable mutations in the JAK/STAT, MAPK, MYC, and chromatin modifying pathways in both Vδ1 and Vδ2 lymphomas. Small molecule inhibitors can be matched to oncogenic targets, thus laying the foundation for therapeutic approaches for this treatment refractory and often deadly disease. Furthermore, MAPK pathway mutations were absent in the Vδ1 γδ MFs but present in Vδ1 PCGDTLs; whether this difference is robust will require investigations of a larger cohort.

Lastly, our studies provide insights into an underappreciated cell type in human skin. In mouse models, γδ T cells play a prominent role in multiple fundamental immunological and epidermal processes. They protect against microbes, tumors, and contribute to epidermal homeostasis^27^. Nonetheless, their functions in humans remains obscure. This may be in part due to their relative scarcity and heterogeneity in human tissue such as the skin.

Interestingly, we have identified clinically relevant γδ T cells in humans via “single-cell” analyses. Regardless of the presence of cancer-causing mutations, these cancers are in essence a monoclonal proliferation of a γδ T cell clone. These studies may have uncovered important tissue-specific roles for specific γδ T cells. For example, the Vγ3Vδ2 subcutaneous tissue-resident T cell that evolves into panniculitic PCGDTLs may play an important role in adipose metabolism, homeostasis, and/or disease^59^.

## Methods

### Study design

This study was reviewed and approved by local institutional review boards and adhered to ethical principles put forth in the Declaration of Helsinki. All relevant ethical regulations for work with human participants were complied with and informed consent was obtained. This study was reviewed and approved in compliance with the Northwestern University Institutional Review Board. Research participants provided informed consent for publication of images. Participating institutions included Northwestern Memorial Hospital, Massachusetts General Hospital, the University of Virginia Hospital and the University of Chicago Hospital. The inclusion criteria were a clinical diagnosis of primary cutaneous lymphomas with confirmation of the γδ T cell receptor either by IHC staining or next-generation sequencing analysis. Across four institutions, we identified 42 cases that met inclusion criteria, and sequenced tissue from 29 patients with available tissue. Analysis of next-generation-sequencing data was blind to clinical outcomes.

### Analysis of clinical characteristics

The cohort was classified based upon morphology of cutaneous lesions, characteristics of the atypical infiltrate on histology, and the presence or absence of cytotoxic cell markers (TIA-1, granzyme B, perforin). Clinical information was obtained from the electronic medical record of each patient when available and is summarized in Supplementary Table 1. Metastatic spread was determined on the basis of positron emission tomography-computed tomography (PET–CT) or computed tomography (CT) imaging highly suspicious for metastatic involvement. One-third of these cases had metastasis proven by biopsy and/or cytology. Antibodies used to determine γδ TCR expression included γ3.20 antibody clone (TCR1153, ThermoFisher Scientific, IL) or H-41 antibody clone (SC-100289, Santa Cruz Biotechnology, TX)^60,61^. Major pathological criteria that were assessed were depth of infiltrate involvement, ulceration, keratinocyte necrosis, and vasculitis. Samples were divided in groups based on depth of skin involvement: (1) epidermal (2) epidermal/dermal, and (3) panniculitic. There was insufficient clinical annotation to distinguish between epidermal vs. epidermal/dermal involvement in five samples. Biopsy specimens were subject to review by expert pathologists (J.G., A.L., and/or A.G.).

### Statistical analysis

Statistical analysis was performed using GraphPad Prism (v8.0.0). Differences in categorical variables between groups utilized Fisher’s Exact Test. Differences in survival were assessed using the log-rank (Mantel Cox) test. A *P* value of < 0.05 was considered statistically significant. The end-point for survival analyses was (1) mortality due to any cause or (2) hematopoietic stem cell transplant (HSCT).

### Patient samples and DNA/RNA isolation

WES, WGS, targeted sequencing, and RNA-Seq were performed on formalin fixed, paraffin embedded (FFPE) blocks or fresh frozen tumor samples (Supplementary Table 3). FFPE samples were obtained by needle cores taken from tumor-rich areas of each block with >80% tumor cells. DNA and RNA were extracted using QIAamp FFPE DNA (Qiagen) or FFPE RNA/DNA Purification Plus (Norgen) kits, respectively. For frozen tumor samples, tumor biopsies were stored immediately in RNAlater (ThermoFisher) and then extracted using AllPrep DNA/RNA Mini (Qiagen).

### WES and WGS sequencing

For WES and WGS, library preparation was performed using KAPA Hyper Prep Kit (Illumina) per the manufacturer’s instructions. For WES, exome capture was performed with IDT xGen Exome Research Panel v1.0. Library pools were loaded onto an Illumina Hiseq in 2 × 150 bp format.

### Targeted sequencing

Library preparation was performed using the TruSight Tumor 170 Kit (Illumina) per manufacturer instructions. Libraries were sequenced on an Illumina Hiseq in 2 × 100 bp format.

### RNA sequencing

RNA-Seq libraries were constructed using SMARTer Stranded Total RNA-Seq Kit (Takara Bio) for FFPE samples and SMART-Seqv4 Ultra Low Input RNA Kit (Takara Bio) for fresh frozen samples followed by NexteraXT DNA Library Prep Kit (Illumina). Samples were sequenced on an Illumina HiSeq with a read length configuration of 150 PE.

### High throughput T cell receptor sequencing and analysis

For seven samples analyzed by TCR-Seq, three 5 μM FFPE sections were utilized. For the one remaining sample, DNA was extracted from RNA*later* stabilized frozen tumor sample using the Qiagen AllPrep DNA/RNA Mini Kit. FFPE samples were shipped to Adaptive Biotechnologies where DNA was extracted. TCRalpha/delta and TCRgamma CDR3 regions were amplified and sequenced using the ImmunoSEQ platform at survey-level resolution (Adaptive Biotechnologies). The resulting data was analyzed using the immunoSEQ Analyzer 3.0 (Adaptive Biotechnologies).

### DNA-sequencing analysis

Twenty patients had their lymphomas analyzed by DNA-Seq including 12 sequenced by WES, 1 by WGS, 5 by both WES and WGS, and 2 by targeted sequencing. For three patients with no DNA-sequencing available, we analyzed the RNA-seq data for SSNVs. For samples with both WES and WGS, WES results were used for SSNV analysis and WGS used for SCNV analysis. Reads were aligned to the human reference genome 19 (hg19) using the Burrow–Wheeler Alignment tool^62^. For single nucleotide variant calling, variants were first called using MuTect^63^. Quality control of variants was performed as previously described^64^. For samples from FFPE tissue, we removed variants with significant strand orientation bias as previously described^65^. To call copy number variants (CNVs), WES data was processed using GATK4CNV^66^. We utilized Patchwork to call CNVs for WGS data^67^. We filtered CNVs which were observed in healthy controls in GnomAD at an allele frequency >10%. Purity of each sample was estimated as described previously^68^, based on the log 2 read ratio of chromosome arm level CNVs. GISTIC2.0 was used to identify statistically significant copy number alterations across the cohort^39^. COSMIC mutational signatures were assessed using MusiCa software^69^.

### RNA-Seq analysis

Reads were aligned to hg19 using STAR^70^. HTseq was utilized to generate gene counts^71^. To identify differentially expressed genes between Vδ1 and Vδ2 lymphomas, we compared three Vδ1 samples (GD6, GD8, GD17) and three Vδ2 samples (GD12, GD13, GD22) that were processed similarly and in the same batch. We identified differentially expressed genes using DE-Seq2, considering genes with an adjusted *P* value <0.05 as significant^18^. To identify enrichment of gene sets in differentially expressed genes, we utilized GSVA software with published gene sets (MSigDB)^22^. GO pathway analysis and CheA transcription factor-binding analysis was applied to statistically significantly differentially expressed genes^23^. To extract TCR sequences from RNA-seq and WGS data, we first employed MiXCR software^14^, which revealed the γδ TCR for 82% of samples with RNA-Seq data. For the remaining samples, we utilized STAR to align the FASTQ files to a custom reference genome of all γδ TCR genes and used HTseq to count the transcripts mapping to each γδ TCR gene. For analysis of published Vδ1 and Vδ2 RNA-Seq, we downloaded FPKMs from the Gene Expression Omnibus (GSE124731) and used Ballgown^72^ to identify differentially expressed transcripts between normal Vδ1 and Vδ2T cells. To identify gene fusion candidates, PRADA software was utilized^31^.

### TCR retroviral transduction

We cloned full-length cDNA of TCRγ and TCRδ chains, separated by self-cleaving T2A sequence, of either the 9C2 TCR^28^ or GD8 TCR into the MSGV retroviral expression vector (Addgene #107226). CD8+ T cells were retrovirally transduced with GD8, 9C2, or FMC63-28Z anti-CD19 CAR as previously described^73^ and sorted on a BD FACSARIA 5 (BD Bioscience) for γδTCR+ cells one day post transduction (Supplementary Fig. 6a). After two days of expansion following sorting, γδ TCR and CD1d-PBS-57 staining was assessed by flow cytometry.

### HEK293 cell transfection

We cloned full-length cDNA of human CD3δ, CD3γ, CD3ε, and CD3ζ subunits, separated by self-cleaving F2A, T2A, and P2A sequences into pLenti CMV Blast empty lentiviral expression vector (w263-1; Addgene #17486). 1383I αβ TCR was a gift from Michael Nishimura and was cloned to the MSGV vector. Cells were transfected with equimolar amounts of CD3 and TCR plasmids with PEI (1 mg/ml). Two days post transfection, CD3, γδ TCR, and CD1d-PBS-57 staining was assessed by flow cytometry.

### Mononuclear cell isolation from skin and subcutaneous tissue

Tissue from five healthy controls undergoing abdominoplasty was obtained. Tissue was washed in PBS, adipose tissue was separated from epidermis and dermis using scissors, and both subcutaneous tissue and epidermis/dermis were digested overnight in a shaker in 60% RPMI and 40% dispase (StemCell Technologies). Epidermis was then peeled from dermis and separately washed in RPMI and filtered through a 70 μM filter. Subcutaneous tissue was filtered with a 100 μM filter, washed with PBS, and centrifuged. Floating adipocytes were removed by pipetting and the cell pellet was resuspended in PBS, and filtered via 70 μM filter. From the resulting cell suspensions, mononuclear cells were isolated via density centrifugation using Isolymph (Fisher Scientific) per manufacturer instructions. The isolated mononuclear cells were then assessed by flow cytometry.

### Tumor cell isolation, analysis, and γδ TCR scRNA-seq

Fresh tumor biopsy obtained from one PCGDTL patient (GD42) was obtained. Tissue was washed in PBS and digested overnight in a shaker in 60% RPMI and 40% dispase (StemCell Technologies). Tissue was washed in RPMI and filtered through a 70 μM filter. Cells were analyzed by flow cytometry for CD3, γδTCR, Vδ1, Vδ2, Vγ9 and live/dead staining. Live, CD3+, γδTCR+ were sorted using a BD FACSAria 5 and scRNA-seq performed via 10× Genomics single cell V(D)J analysis per the manufacturer’s instructions. To amplify γδ TCR transcripts, outer and inner enrichment primers targeting TRD and TRG gene constant regions (sequences listed below) were designed similar to the approach to αβ TCR transcript amplification, and all steps were performed per manufacturer instructions with the modification of replacing αβ TCR primers with γδ TCR primers, listed below.

TRD outer: 5′-GCTTGACAGCATTGTACTTCC-3′

TRD inner: 5′-GACAAAAACGGATGGTTTGG-3′

TRG outer: 5′-CATGTATGTGTCGTTAGTCTTCATG-3′

TRG inner: 5′-AGGAAGAAAAATAGTGGGCTTG-3′

### Flow cytometry

Cells were resuspended in 100 μl of PBS with 2% FBS prior to staining. For APC-CD1d-PBS-57 tetramer (NIH Tetramer Core Facility) staining, 1 μl was added and cells were incubated at room temperature for 30 min protected from light. Antibodies used include PB-CD3 (1:100, Biolegend, OKT3, Cat #317314), PE-Cy7-Vδ1 (1:100, Invitrogen, TS8.2, Cat #25-5679-42), PE-Vδ2 (1:100, Miltenyi Biotech, 123R3, Cat #130-095-796), FITC-γδ TCR (1:50, Invitrogen, 5A6.E9, Cat #MHGD01-4), and APC-Vγ9 (1:100, Biolegend, B3, Cat #331310). 7-AAD (ThermoFisher, Cat #A1310) was used to discriminate living and dead cells. Flow cytometry was performed on a BD LSR II (BD) and data was analyzed using FlowJo (v10.5.3).

### TCR structural modeling

An initial model of the GD8 TCR was generated with the Modeller program^74^, version 9.15, using the x-ray structure of 9C2 TCR (Protein Data Bank code: 4LHU) as template. The 9C2 TCR was selected as modeling template as it shares delta (Vδ1) and gamma (Vγ5) germline genes with the GD8 TCR^28^. The modeled GD8 structure was superimposed onto the 9C2 TCR structure bound to CD1d-α-galactosylceramide (α-GalCer), followed by docking using the TCRFlexDock algorithm^75^ to refine the model of the GD8–α-GalCer–CD1d complex. TCRFlexDock was developed in the Rosetta modeling framework^76^ and was previously used to model TCR interactions with CD1 and MR1^77^, in addition to peptide–MHC complexes. It uses an iterative combination of rigid-body movements, side chain sampling, and loop conformational sampling during its docking procedure. Docking models were scored using the Rosetta scoring function^78^, and interface score, which represents intermolecular interaction energy, was used to select the refined model.

### Reporting summary

Further information on research design is available in the Nature Research Reporting Summary linked to this article.

## Supplementary information

Supplementary Information

Description of Additional Supplementary Information

Supplementary Data 1

Supplementary Data 2

Supplementary Data 3

Supplementary Data 4

Reporting Summary

## Data Availability

The DNA and RNA sequencing data have been deposited in the database of Genotypes and Phenotypes (dbGaP) under the accession code [https://www.ncbi.nlm.nih.gov/projects/gap/cgi-bin/study.cgi?study_id=phs001969.v1.p1] and are available. All the other data supporting the findings of this study are available within the article, source data, and supplementary files or from the corresponding author upon reasonable request. A reporting summary for this article is available as a Supplementary Information file.
