# Supplementary material for: Cellular origins and genetic landscape of cutaneous gamma delta T cell lymphomas
Source: Nat Commun. 2020 Apr 14;11:1806. doi: 10.1038/s41467-020-15572-7 (PMC7156460; doi:10.1038/s41467-020-15572-7)
Supplement: Supplementary file 1 — Supplementary Information [file 41467_2020_15572_MOESM1_ESM.pdf]

Supplementary Information for:

Cellular Origins and Genetic Landscape of Cutaneous Gamma Delta T Cell Lymphomas

Daniels et al

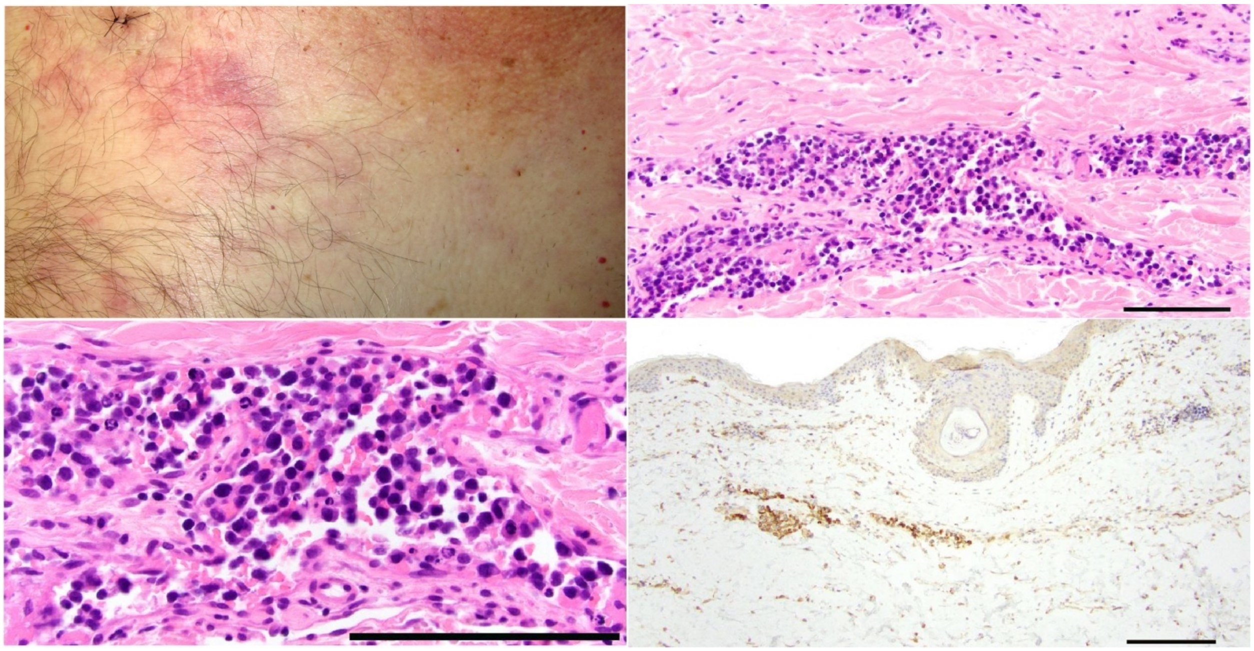

Supplementary Figure 1. Intravascular presentation of γδ T cell Lymphoma. Clinical photograph (top left), hematoxylin and eosin staining (top right, 40x and bottom left, 100x), and γδ T cell receptor immunostaining (10x, bottom right) of the lone intravascular case. Scale bar represents 100 μm in the top right panel, 50 μm in the bottom left panel, and 200 μm in the bottom right panel.

a

	Bulk RNA-seq	WGS	scRNA-seq	Flow cytometry
Vδ2: CACDTGVTAQLFF	Yes	Yes	Yes	Vδ2+
Vγ3: CATWDHTTGWFKIF	Yes	Yes	Yes	Vγ9-

b

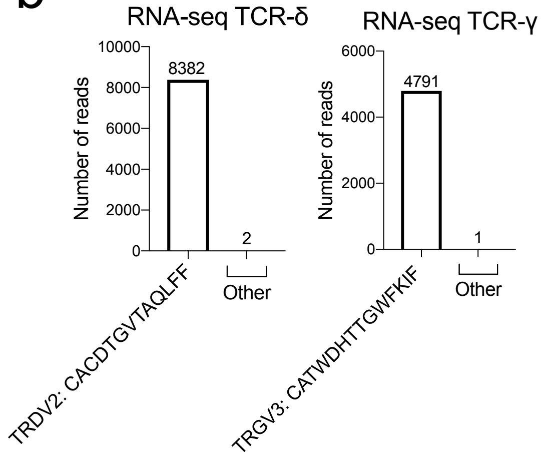

c

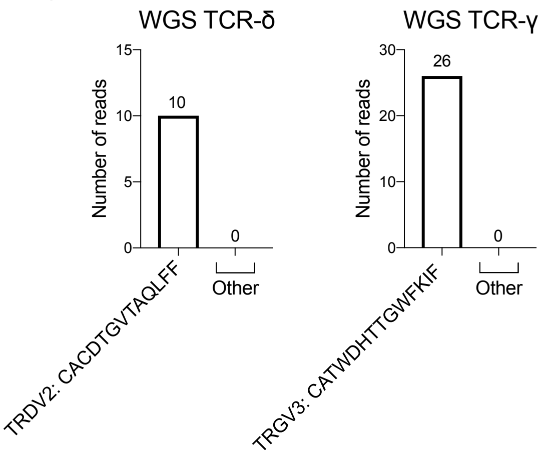

d

Cell Barcode	Chain	V gene	CDR3	Reads
CACAGTAAGACTGTAA-1	TRD	TRDV2	CACDTGVTAQLFF	78046
CACAGTAAGACTGTAA-1	TRG	TRGV3	CATWDHTTGWFKIF	1344

e

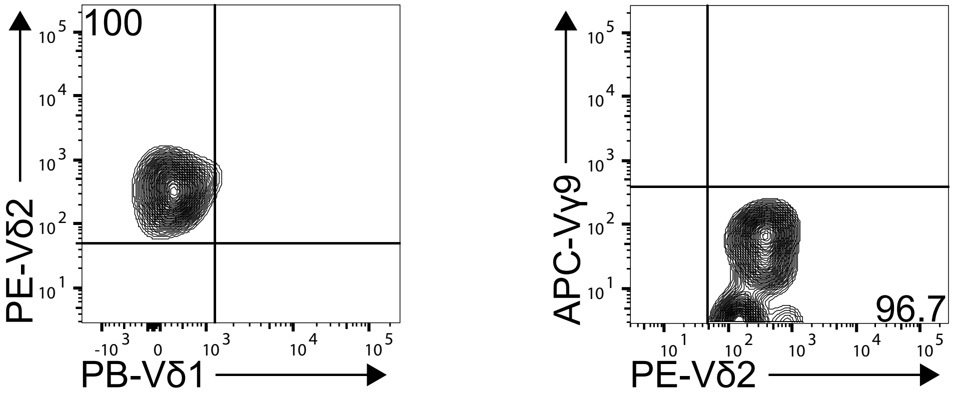

Supplementary Figure 2. Analysis of T cell receptor usage in sample GD42 by multiple orthogonal approaches. (a) Table showing concordant results obtained by RNA-sequencing, whole genome sequencing (WGS), single cell RNA sequencing (scRNA-seq), and flow cytometry. (b) T cell receptor (TCR) clonotypes determined by bulk RNA-sequencing. (c) TCR clonotypes determined by WGS. (d) scRNA-sequencing demonstrates pairing of Vδ2 and Vγ3 chains in the same cell. (e) Flow cytometric analysis of TCR chain usage in cells from fresh tumor, gated on live, CD3+, γδ TCR+ cells.

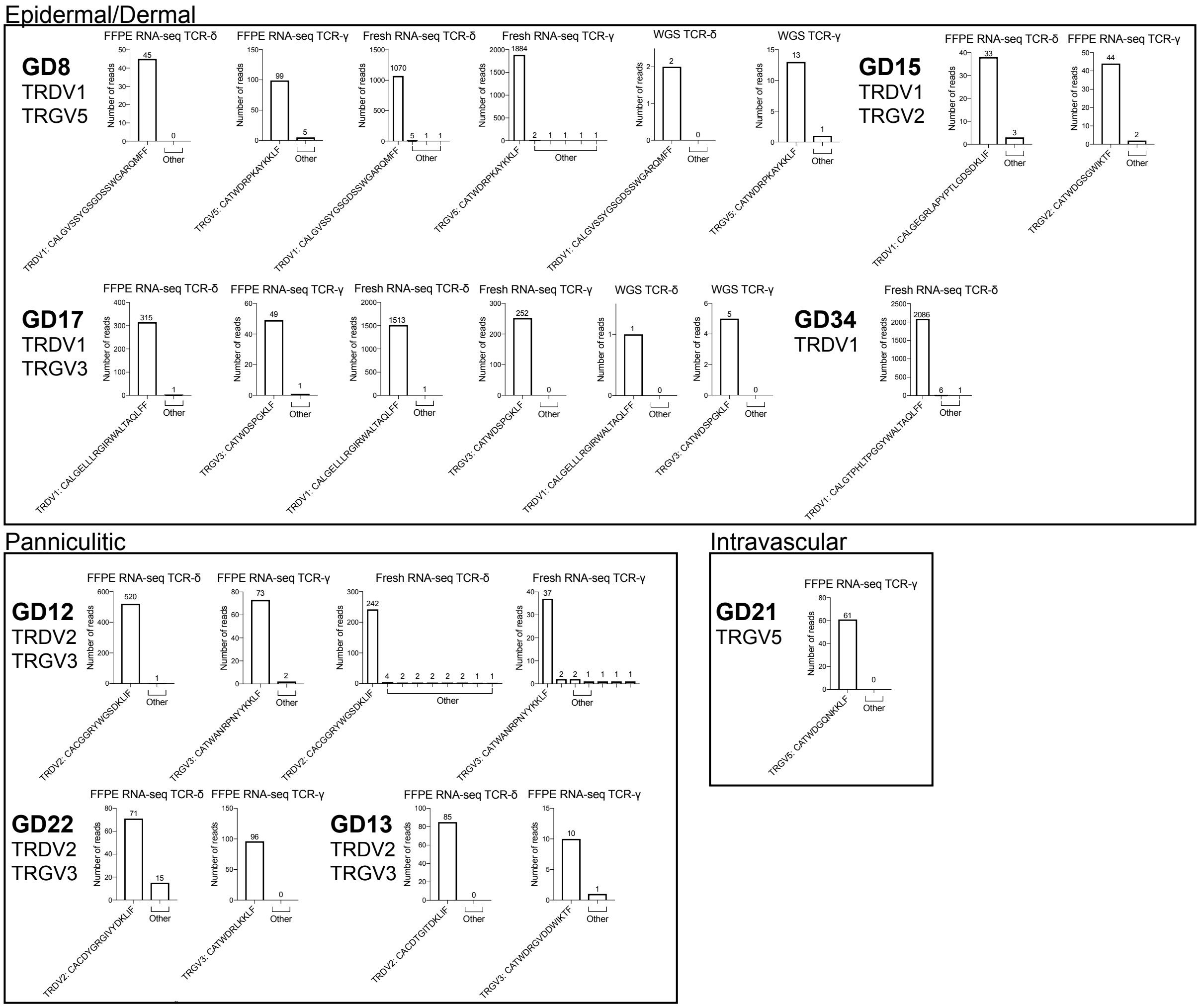

Supplementary Figure 3. T cell receptor gene usage in CGDTL samples identified by RNA-seq and whole genome sequencing. Bar plots indicating frequency of reads supporting productive TCR γ and δ clonotypes, grouped by disease histology. For each sample, the most common γ or δ clonotype is indicated, and others (if detected) are denoted as “other”. In all cases where multiple sequencing results could be assessed, results across different sequencing modalities or sample preparation were concordant each time (GD8, GD12, GD17). For sample GD17, two TRDV1 clonotypes present in approximately equal frequencies that differed by a single amino acid were combined.

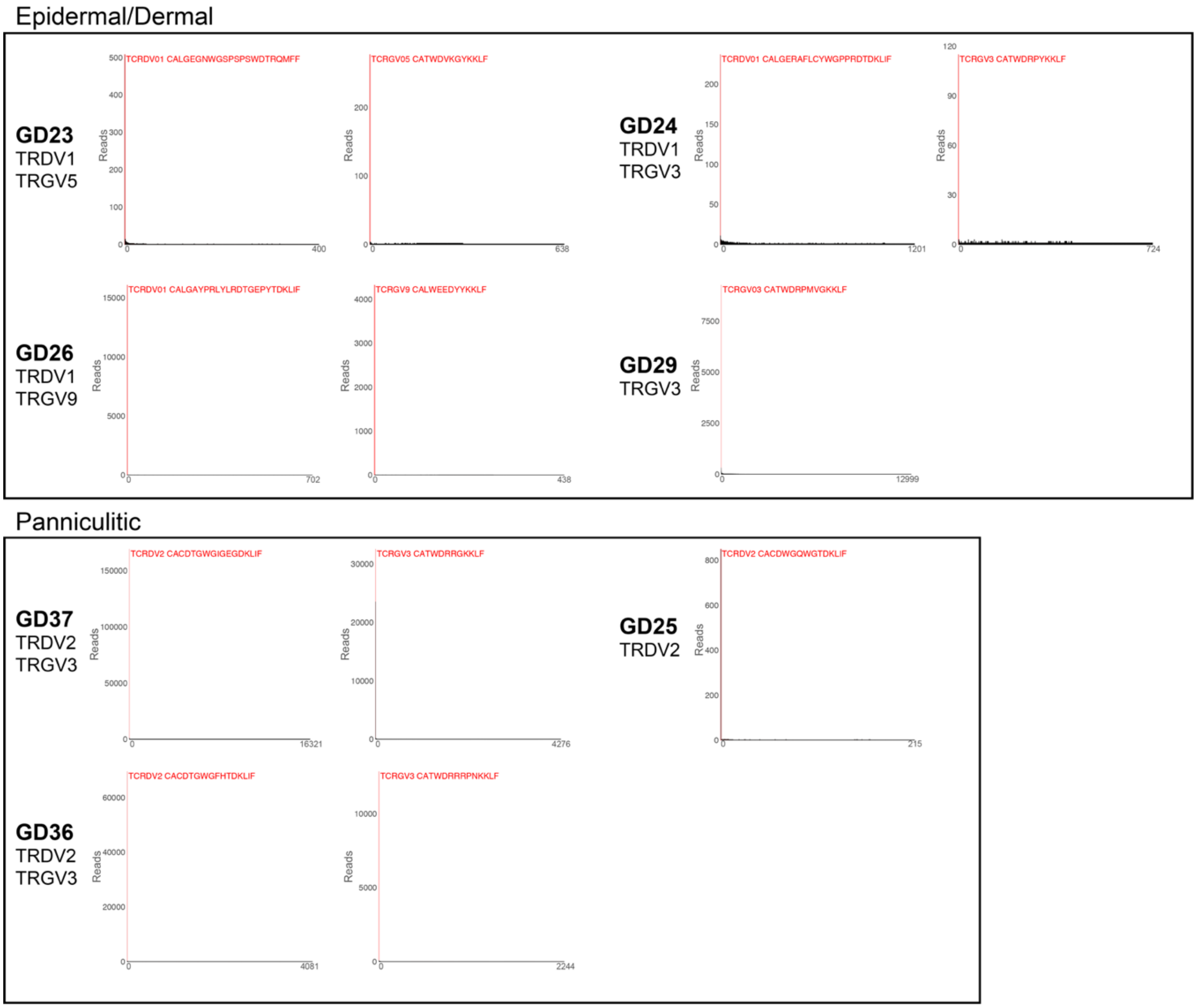

Supplementary Figure 4. T cell receptor gene usage in CGDTL samples identified by high throughput TCR-Seq. Bar plots indicate frequency of reads supporting TCR γ and δ clonotypes, grouped by disease histology. X-axis indicates each unique clonotype detected via TCRδ/α or TCRγ sequencing. For each sample, the most common δ or γ clonotype is indicated in red.

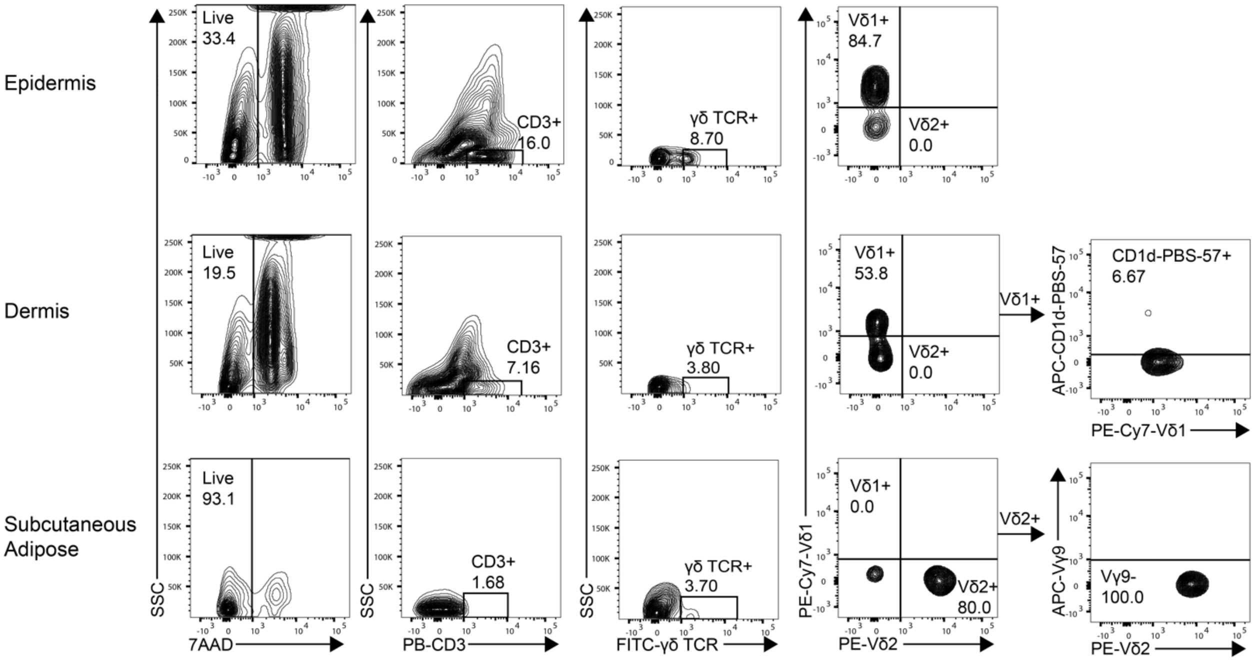

Supplementary Figure 5. Gating strategy to identify γδ T cells in normal human skin. Gating strategy and identification of γδ T cells representative of 5 donors. Representative examples of Vδ1 and Vδ2 cell frequencies (related to Fig. 1c,d), CD1d-PBS-57+ frequency in epidermis and dermis (related to Fig. 4c) and Vγ9+ frequencies of Vδ2 cells in subcutaneous adipose tissue (related to Fig. 4b) are shown.

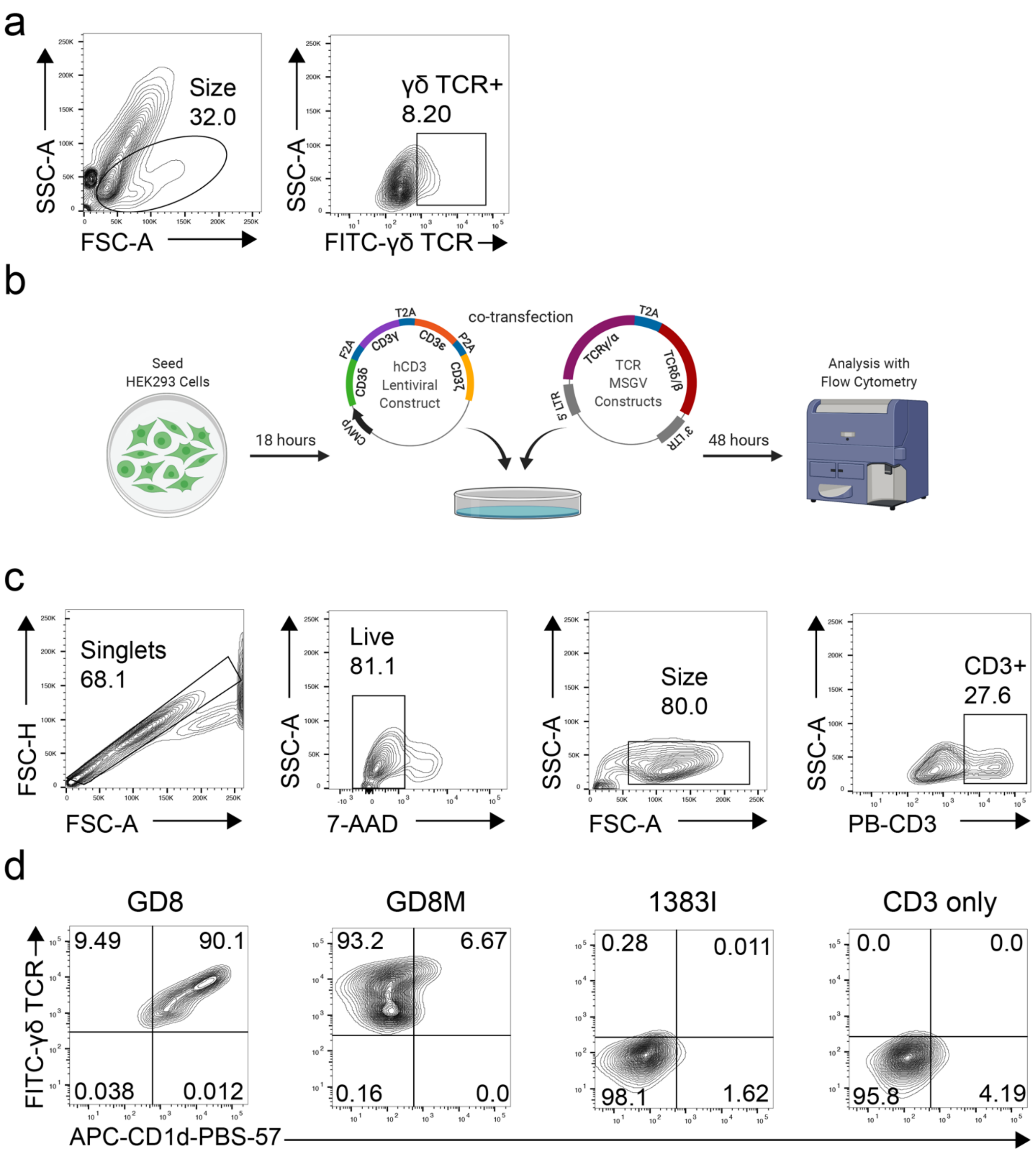

Supplementary Figure 6. Flow cytometry analysis of γδ TCR expression in primary T cells and CD3 co-transfected HEK293 cells. (a) Gating strategy to sort γδ TCR transduced CD8+ T cells following retroviral transduction (related to Fig. 4d). (b) Experimental scheme of co-transfection experiments in HEK293 cells. (c) Gating strategy to identify CD3+ cells following transfection of HEK293 cells (Related to Fig. 4f,g). (d) Representative contour plots of γδ TCR and CD1d-PBS-57 staining gated on CD3+ cells.

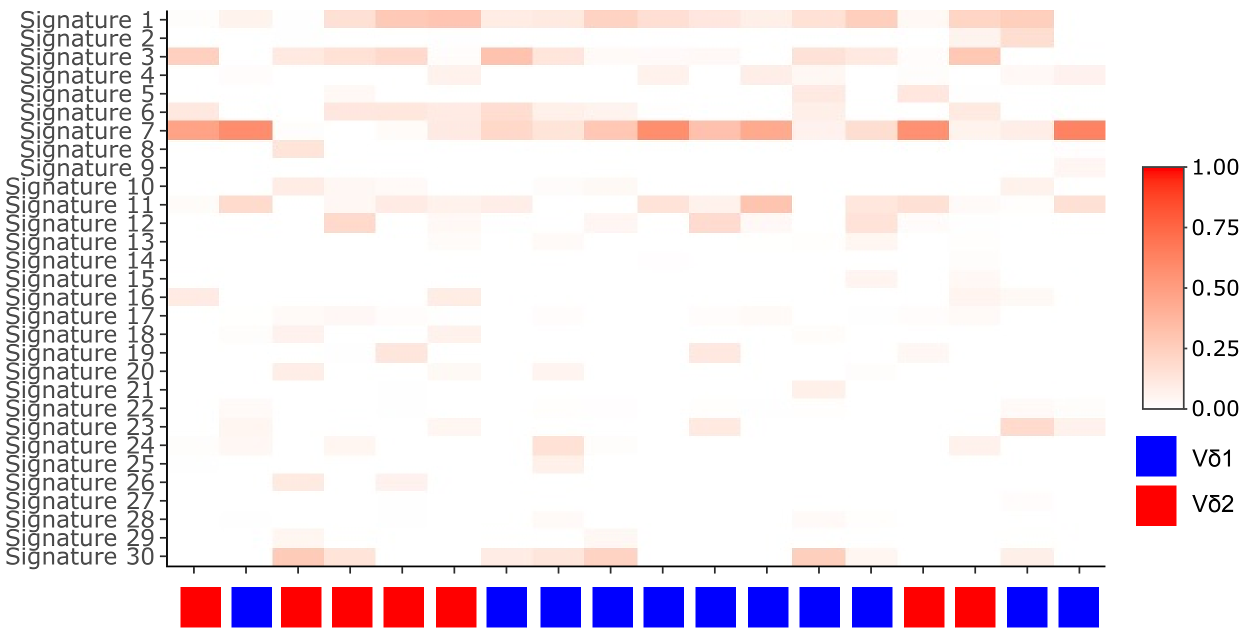

Supplementary Figure 7. Mutational signatures of CGDTL. Contribution of COSMIC mutational signatures towards mutations across 17 whole exome sequencing PCGDTL and γδ MF samples. Columns represent samples, and scale indicates fraction of mutations corresponding to each mutational signature among each sample.

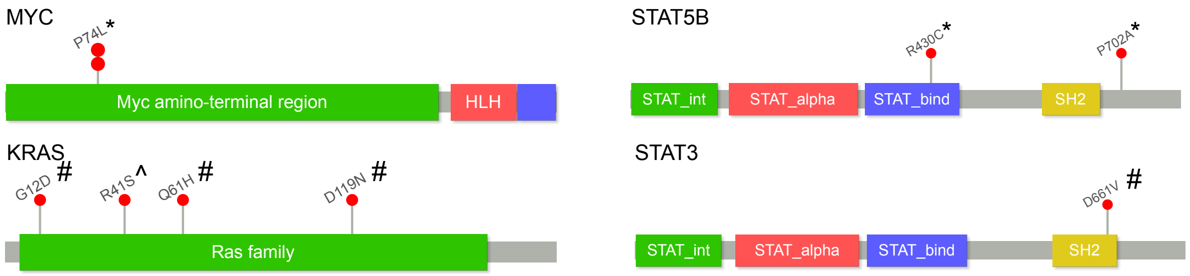

Supplementary Figure 8. Putative oncogenic mutations affecting MYC, MAPK, and JAK/STAT signaling in PCGDTL and γδ MF. Schematics highlighting missense mutations in *MYC*, *KRAS*, *STAT5B*, and *STAT3*. * indicates mutations previously reported in other cancers. # indicates mutations previously validated as oncogenic. ^ variant of uncertain significance.

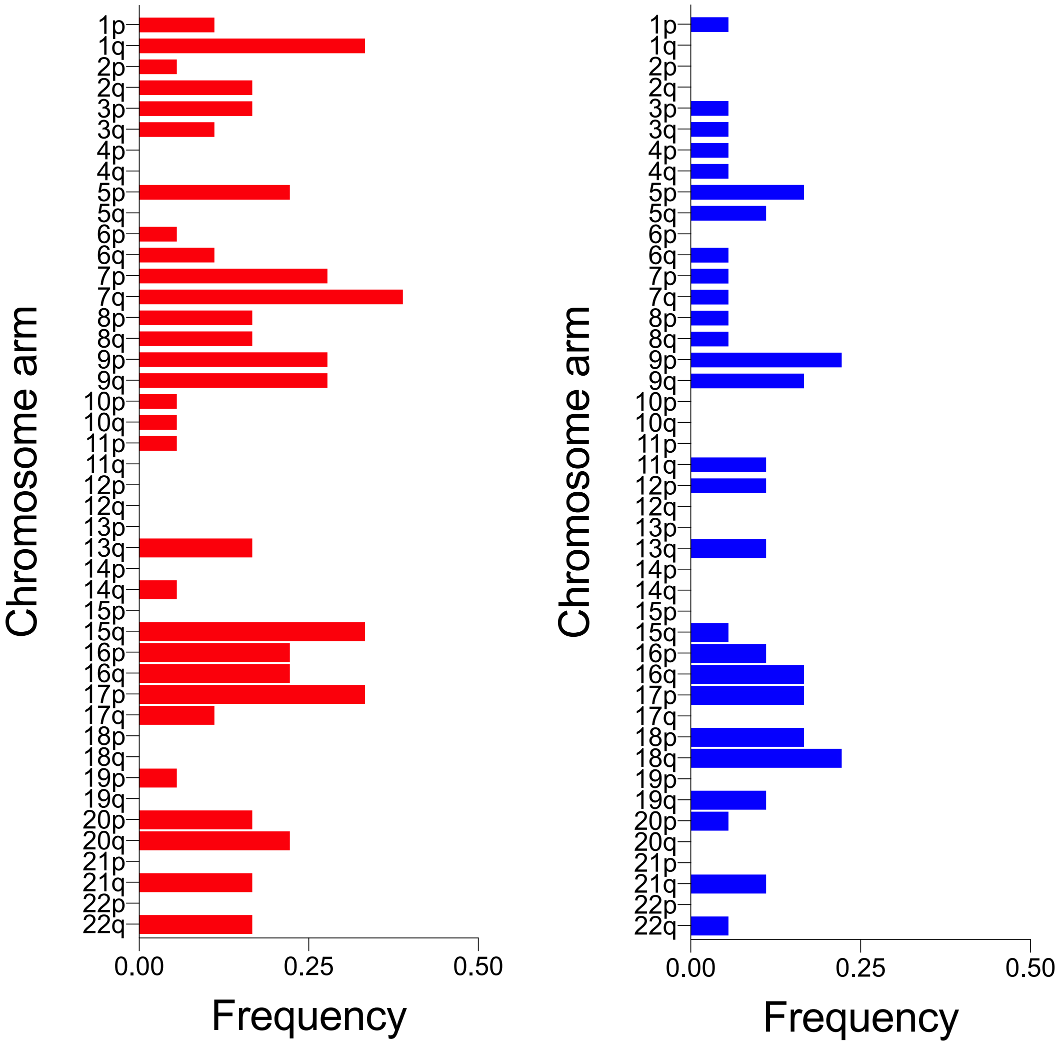

Supplementary Figure 9. Frequency of chromosome arm level copy number alterations in PCGDTL and γδ MF. Frequency of amplifications (left) and deletions (right) involving chromosome arms.

Case	Clinical Diagnosis	Pathology	Phenotype Switch	Race	Lesion Type	Ulcerated Lesions	B Symptoms	Lymph Nodes	Extranodal Localization	Follow up (months)	Status at follow-up	Cause of Death
GD1	PCGDTL	E/D		C	plaque	y	n	n		4	D*	HSCT
GD2	PCGDTL	E/D			plaque	n	y	y		32	D+	
GD3	γδ MF	E/D	y		patch	n	n	n		59	D+	
GD4	γδ MF	E/D	y	C	patch	n	n	n	Intestine*	89	D+	PCGDTL
GD5	PCGDTL	E/D		C	tumor	n	n	y	Liver*, CNS*	4	D*	PCGDTL
GD6	PCGDTL	E/D				y	n			31	D+	
GD7	PCGDTL	E/D		C	tumor	y	n	n		4	D*	HSCT
GD8	PCGDTL	E/D		C	tumor	y	y	n		10	A-	
GD9	PCGDTL	Panniculitic		A	tumor	n	n	n		36	D+	
GD10	PCGDTL	E/D		C	plaque	y	n	n		12.5	D+	
GD11	PCGDTL	E/D		C	tumor	y	n	y		30	A+	
GD12	PCGDTL	Panniculitic		C	patch	y	n	n		5	D*	HSCT
GD13	PCGDTL	Panniculitic		C	patch	y	n	n		19.5	D+	
GD14	PCGDTL	E/D		C	tumor	y	n	n	spleen, intestine	1.5	A+	
GD15	γδ MF	E/D	y	C	plaque	n	y	n	CNS*	180	D+	PCGDTL
GD16	γδ MF	E/D	y	C	patch	n	n	n		33	D+	PCGDTL
GD17	γδ MF	E/D	y	C	patch	n	n	n		146	D+	PCGDTL
GD18	γδ MF	E		C	patch	n	n	n		16	A-	
GD19	γδ MF	E		C	plaque	n	n	n		255	A-	
GD20	γδ MF	E	y	C	plaque	n	n	n		107	D*	HSCT
GD21	IVGDTL	IV		C	patch	n	y	n	spleen	5	D*	HSCT
GD22	PCGDTL	Panniculitic		C	tumor					1	D+	
GD23	γδ MF	E		C	plaque	n	n	n		16	A+	
GD24	γδ MF	E		A	plaque	n	n	n		15.5	A+	
GD25	PCGDTL	Panniculitic		C	tumor	y	y	y		7.5	D+	
GD26	PCGDTL	E/D		C	plaque	y	n	n	intestine	1.5	D+	PCGDTL
GD27	γδ MF	E		C	plaque	n	n	n		75	A+	
GD28	γδ MF	E			patch	n	n	n		39.5	A-	
GD29	γδ MF	E/D		A	plaque	n	n			410	A+	
GD30	PCGDTL	E/D		A	plaque	y	y			17	A+	
GD31	γδ MF	E		H	patch	n	n	n		18	A+	
GD32	γδ MF	E		C	plaque	n	n	n		36.5	A+	
GD33	PCGDTL	Panniculitic		C	plaque	n	y			7	D+	Sepsis
GD34	γδ MF	E/D		C	tumor	n	n	n		169	A+	
GD35	PCGDTL	E/D								0		
GD36	PCGDTL	Panniculitic		A	tumor	n	n			36	A+	
GD37	PCGDTL	Panniculitic		C	tumor	n	n	n		18	D+	PCGDTL
GD38	PCGDTL	Panniculitic		C	tumor	y	y	n	vertebral bone	3	D+	ICH
GD39	PCGDTL	Panniculitic		C	tumor	n	y	y	liver, spleen	77	D	HLH
GD40	PCGDTL	Panniculitic		C	tumor	y	y	y		11	A+	
GD41	PCGDTL	Panniculitic		C	tumor	n	y	y	liver, spleen, CNS	2	D	Sepsis
GD42	PCGDTL	Panniculitic		C	Tumor	y	y	n		0		

Supplementary Table 1: Detailed clinical information of PCGDTL and γδ MF cohort. Characteristics such as lesion type, ulcerations, B symptoms, and lymph node

involvement are from the initial clinical presentation. HLH and extranodal localization are from any time point during clinical follow-up. Blank boxes denote a characteristic that is either not applicable to that sample or information that was unable to be obtained from the medical record. * in Extranodal Localization column denotes biopsy or cytology proven metastatic spread. Primary Cutaneous γδ T cell Lymphoma (PCGDTL), γδ Mycosis Fungoides (γδ MF), Intravascular γδ T cell Lymphoma (IVGDTL), Epidermis +/- Dermis (E/D), Epidermis (E), Caucasian (C), African American (A), Hispanic (H), yes (y), no (n), Central Nervous System (CNS), Alive with Disease (A+), Alive without Disease (A-), Dead with Disease (D+), Hematopoietic Stem Cell Transplant (D*), Hemophagocytic Lymphohistiocytosis (HLH), Intra-cerebral Hemorrhage (ICH).

Case	Pathology	CD3	CD2	CD7	CD56	CD5	CD4	CD8	γδTCR	Granzyme B	Perforin	TIA-1
GD1	E/D	P		N	P	N	N	N	P	P		P
GD2	E/D	P					N	N	P	N		P
GD3	E/D						N	N	P			
GD4	E/D	P	P	P	N	N	P	N	P	P		P
GD5	E/D	P	N	P	P	N	N	N	P			N
GD6	E/D	P		P		N	N	P	P			P
GD7	E/D	P		N	P	N	N	N	P			P
GD8	E/D	P	N	N	N	P	N	N	P			P
GD9	Panniculitic							P	P			
GD10	E/D	P	P	N	N	P	N	N	P	N		P
GD11	E/D	P		N		N	N	N	P	N		N
GD12	Panniculitic	P	P	N	P	N	N	N	P			P
GD13	Panniculitic	P				N		N	P	P		N
GD14	E/D	P	P	N		P	N	N	P			N
GD15	E/D	P		N		N	P	N	N			
GD16	E/D	P	N		N	N	N	N	P	P		P
GD17	E/D	P	P	N	N	P	P	N	P	N		N
GD18	E	P		N	N	N	N	P	P	N		P
GD19	E				N	N	N	P	P			
GD20	E	P	N	N	N	N	N	N	P	N*		N*
GD21	IV	P	P	N	N	P	N	N	P			P
GD22	Panniculitic	P			P	N	N	N	P			P
GD23	E	P					N	N	P			N
GD24	E	P		N			N	N	P			
GD25	Panniculitic	P			N	N	P		P			
GD26	E/D	P			N		N	N	P			N*
GD27	E	P					P	N	P			N
GD28	E			N	N		N	N	P			P
GD29	E/D	P	P	P	N	N	N	N	P			P
GD30	E/D	P	N	N		N	P	N	P	P		P
GD31	E	P		P	P	N	N	P	P	P		P
GD32	E		N	P	N	N	N	N	P			P
GD33	Panniculitic								P	P		
GD34	E/D	P		N	N	N	P	N	P			N
GD35	E/D	P		N	N				P			
GD36	Panniculitic	P	P	N	P	N	N	N	P		P	P
GD37	Panniculitic	P	P	N		N	N	N	P			
GD38	Panniculitic	P	P	N	P	N	N	N	P	P	P	
GD39	Panniculitic	P			P	P	N	P	P	P		P
GD40	Panniculitic	P			P		N	P	P	P		P
GD41	Panniculitic	P	P	N	P	N	N	N	P			P
GD42	Panniculitic	P	N	N	P	N	N	N	P			P

Supplementary Table 2: Histologic phenotypes in PCGDTL and γδ MF. * denotes cases that were initially negative at diagnosis but tested positive at a later time. Epidermal (E), Epidermal and/or Dermal (E/D), Intravascular (IV), Positive (P), Negative (N).

Sample	Histology	WES	Source	WGS	Source	RNA-seq	Source	TCR-seq	Source	Targeted	Source
GD3	E/D	Yes	FFPE								
GD4	E/D	Yes	FFPE								
GD5	E/D	Yes	FFPE								
GD6	E/D	Yes	FFPE			Yes	FFPE				
GD7	E/D	Yes	FFPE								
GD8	E/D	Yes	FFPE	Yes	Fresh	Yes	FFPE, Fresh				
GD9	P	Yes	FFPE								
GD10	E/D	Yes	FFPE								
GD12	P	Yes	FFPE	Yes	Fresh	Yes	FFPE, Fresh				
GD13	P	Yes	FFPE			Yes	FFPE				
GD14	E/D	Yes	FFPE	Yes	Fresh						
GD15	E/D	Yes	FFPE			Yes	FFPE				
GD17	E/D	Yes	FFPE	Yes	Fresh	Yes	FFPE, Fresh				
GD18	E					Yes	FFPE				
GD21	IV					Yes	FFPE				
GD22	P					Yes	FFPE				
GD19	E							HTS	Slides		
GD23	E							HTS	Slides		
GD24	E							HTS	Slides		
GD25	P							HTS	Slides		
GD26	E/D							HTS	Slides		
GD29	E/D							HTS	Fresh		
GD34	E/D	Yes	Fresh	Yes	Fresh	Yes	Fresh				
GD36	P							HTS	Slides	Yes	FFPE
GD37	P							HTS	Slides	Yes	FFPE
GD39	P	Yes	FFPE								
GD40	P	Yes	FFPE								
GD41	P	Yes	FFPE								
GD42	P			Yes	Fresh	Yes	Fresh	scRNA			

Supplementary Table 3: PCGDTL and γδ MF samples interrogated by next generation sequencing. formalin fixed, paraffin embedded sample (FFPE), epidermal/dermal disease (E/D), epidermal disease (E), panniculitic disease (P), intravascular disease (IV), high-throughput sequencing (HTS), scRNA (single cell RNA-seq).

Sample	Histology	V delta	Delta CDR3	Counts	V gamma	Gamma CDR3	Counts
GD8 FFPE	E/D	TRDV1	CALGVSSYGSGDSSWGARQMFF	45	TRGV5	CATWDRPKAYKKLF	99
GD8 Fresh	E/D	TRDV1	CALGVSSYGSGDSSWGARQMFF	1070	TRGV5	CATWDRPKAYKKLF	1884
GD12 FFPE	P	TRDV2	CACGGRYWGSDKLIF	520	TRGV3	CATWANRPNYYKKLF	73
GD12 Fresh	P	TRDV2	CACGGRYWGSDKLIF	242	TRGV3	CATWANRPNYYKKLF	37
GD13 FFPE	P	TRDV2	CACDTGITDKLIF	85	TRGV3	CATWDRGVDDWIKTF	10
GD15 FFPE	E/D	TRDV1	CALGEGRLAPYPTLGDSDKLIF	38	TRGV2	CATWDGSGWIKTF	44
GD17 FFPE	E/D	TRDV1	CALGELLLRGIRWALTAQLFF	158	TRGV3	CATWDSPGKLF	49
GD17 Fresh	E/D	TRDV1	CALGELLLRGIRWALTAQLFF	1513	TRGV3	CATWDSPGKLF	252
GD21 FFPE	IV				TRGV5	CATWDGQNKKLF	61
GD22 FFPE	P	TRDV2	CACDYGRGIVYDKLIF	71	TRGV3	CATWDRLKKLF	96
GD34 Fresh	E/D	TRDV1	CALGTPHLTPGGYWALTAQLFF	2086			
GD42 Fresh	P	TRDV2	CACDTGVTAQLFF	8382	TRGV3	CATWDHTTGWFKIF	4791
GD8 WGS	E/D	TRDV1	CALGVSSYGSGDSSWGARQMFF	2	TRGV5	CATWDRPKAYKKLF	13
GD17 WGS	E/D	TRDV1	CALGELLLRGIRWALTAQLFF	1	TRGV3	CATWDSPGKLF	5
GD42 WGS	P	TRDV2	CACDTGVTAQLFF	10	TRGV3	CATWDHTTGWFKIF	26

Supplementary Table 4: PCGDTL and γδ MF TCRs determined by MixCR. Formalin fixed paraffin embedded (FFPE), whole genome sequencing (WGS), epidermal/dermal disease (E/D), panniculitic disease (P), intravascular disease (IV).

Sample	Histology	V delta	Counts	V gamma	Counts
GD6	E/D	TRDV1	9	TRGV5	11
GD18	E			TRGV3	9

Supplementary Table 5: PCGDTL and γδ MF TCRs determined by RNA-seq reads aligned to γδ TCR genes. Epidermal/dermal disease (E/D), epidermal disease (E).

Sample	Histology	V delta	Top delta CDR3	Counts	V gamma	Top gamma CDR3	Counts
GD23	E	TRDV1	CALGEGNWGSPSPSWDTRQMFF	508	TRGV5	CATWDVKGYKKLF	277
GD24	E	TRDV1	CALGERAFLCYWGPPRDTDKLIF	237	TRGV3	CATWDRPYKKLF	115
GD25	P	TRDV2	CACDWGQWGTDKLIF	849			
GD26	E/D	TRDV1	CALGAYPRLYLRDTGEPYTDKLIF	16082	TRGV9	CALWEEDYYKKLF	4325
GD36	P	TRDV2	CACDTGWGFHTDKLIF	69519	TRGV3	CATWDRRRPNKKLF	12850
GD37	P	TRDV2	CACDTGWGIGEGDKLIF	169138	TRGV3	CATWDRRGKKLF	32543
GD29	E/D				TRGV3	CATWDRPMVGKKLF	9264

Supplementary Table 6: PCGDTL and γδ MF TCRs determined by high-throughput T cell receptor sequencing. Epidermal/dermal disease (E/D), epidermal disease (E), panniculitic disease (P).

Term	P value	Adjusted P value
cytokine-mediated signaling pathway (GO:0019221)	9.09E-14	1.33E-10
cellular response to interferon-gamma (GO:0071346)	1.47E-09	7.51E-07
interferon-gamma-mediated signaling pathway (GO:0060333)	1.54E-09	7.51E-07
type I interferon signaling pathway (GO:0060337)	1.39E-08	4.07E-06
cellular response to type I interferon (GO:0071357)	1.39E-08	4.07E-06
positive regulation of T cell apoptotic process (GO:0070234)	2.63E-06	0.000643
inflammatory response (GO:0006954)	6.74E-06	0.001413
superoxide metabolic process (GO:0006801)	9.31E-06	0.001707
chemokine-mediated signaling pathway (GO:0070098)	1.18E-05	0.001726
antigen processing and presentation of exogenous peptide antigen via MHC class I (GO:0042590)	1.13E-05	0.001726

Supplementary Table 7: Top 10 pathways upregulated by Gene Ontology (GO) analysis in Vδ2 lymphomas. GO terms enriched in Vδ2 samples compared to Vδ1 samples.

Term	P value	Adjusted P value
HALLMARK_INTERFERON_ALPHA_RESPONSE	0.00041515	0.02075745
HALLMARK_INTERFERON_GAMMA_RESPONSE	0.00164826	0.0412066
HALLMARK_IL6_JAK_STAT3_SIGNALING	0.07280047	0.73562835
HALLMARK_E2F_TARGETS	0.0882754	0.73562835
HALLMARK_ADIPOGENESIS	0.13194984	0.82915858
HALLMARK_FATTY_ACID_METABOLISM	0.1628217	0.82915858
HALLMARK_G2M_CHECKPOINT	0.17332192	0.82915858
HALLMARK_ALLOGRAFT_REJECTION	0.19320608	0.82915858
HALLMARK_COMPLEMENT	0.20922718	0.82915858
HALLMARK_INFLAMMATORY_RESPONSE	0.23641507	0.82915858

Supplementary Table 8: Top 10 hallmark gene sets enriched by Gene Set Variation Analysis (GSVA) in Vδ2 lymphomas. Molecular Signatures Database hallmark terms enriched in Vδ2 samples compared to Vδ1 samples.

Term	P.value	Adjusted.P.value
IRF1_21803131_ChIP-Seq_MONOCYTES_Human	4.46E-08	2.69E-05
RELA_24523406_ChIP-Seq_FIBROSARCOMA_Human	3.57E-07	0.000108
IRF8_27001747_Chip-Seq_BMDM_Mouse	3.53E-05	0.007108
NR1H3_23393188_ChIP-Seq_ATHEROSCLEROTIC-FOAM_Human	7.4E-05	0.011178
IRF8_21731497_ChIP-ChIP_J774_Mouse	0.001107	0.133723
IRF8_22096565_ChIP-ChIP_GC-B_Mouse	0.003414	0.252099
MYB_21317192_ChIP-Seq_ERMYB_Mouse	0.003682	0.252099
GATA6_21074721_ChIP-Seq_CACO-2_Mouse	0.002954	0.252099
SCL_21571218_ChIP-Seq_MEGAKARYOCYTES_Human	0.003756	0.252099
VDR_24787735_ChIP-Seq_THP-1_Human	0.005091	0.307498

Supplementary Table 9: Top 10 transcription factor binding motifs enriched in genes differentially expressed in Vδ2 lymphomas by CheA analysis. CheA transcription factor binding analysis results for genes significantly upregulated (adjusted P value < 0.05, DESeq2) in Vδ2 lymphomas.

Case	Treatments prior to development of clinical cytotoxicity	Treatments after the development of clinical cytotoxicity	Status
GD3	Triamcinolone: PR<br>NB-UVB: PR	NB-UVB: NR, DP	D+
GD4	Eucerin: PR<br>NB-UVB: PR<br>PUVA: PR<br>Bexarotene: PR	NB-UVB: NR, DP<br>Lenalidomide: NR, DP<br>Methotrexate: NR, DP<br>Gemcitabine: PR, DP<br>Romidepsin: NR, DP<br>Nitrogen Mustard: NR, DP	D+
GD15	Bexarotene: PR<br>Interferon: PR<br>Nitrogen Mustard: PR	Methotrexate: NR, DP<br>Romidepsin: NR, DP<br>TSEBT: NR, DP<br>Spot Radiation: NR, DP	D+
GD16	Clobetasol: PR<br>Acitretin: PR<br>NB-UVB: PR	Acitretin: NR, DP<br>NB-UVB: NR, DP<br>Bexarotene: NR, DP<br>TSEBT: PR, DP<br>Spot Radiation: PR, DP<br>Interferon: NR, DP	D+
GD17	Triamcinolone: CR<br>PUVA: CR<br>Bexarotene: CR<br>Mechlorethamine Gel: PR	Triamcinolone: NR, DP<br>Bexarotene: NR, DP<br>Methotrexate: NR, DP<br>Brentuximab: NR, DP<br>Gemcitarabine: NR, DP<br>Miragen: NR, DP<br>Doxorubicine: NR, DP<br>Spot Radiation: PR, DP	D+
GD20	Triamcinolone: PR, DP<br>NB-UVB: PR, DP<br>Bexarotene: PR, DP<br>Interferon: PR, DP	Mogalizumab: NR, DP<br>Gemcitabine: NR, DP<br>Denileukin Diftitox: PR<br>Stem Cell Transplant: CR	D*

Supplementary Table 10: Therapies in γδ MF patients with PCGDTL-like progression. Narrow band ultraviolet B (NB-UVB), psoralen and ultraviolet A (PUVA), total skin electron beam therapy (TSEBT), partial response (PR), complete response (CR), disease progression (DP), no response (NR), deceased with active disease (D+), hematopoietic stem cell transplantation (D*).

Sample	Diagnosis	Predominant Skin Layers Involved	History of Celiac Disease	Initial Skin Stage	Number of Lesions	Ulcerated	Time to Initial Imaging (months)	Initial Imaging	Time to Follow-up Imaging (months)	Follow-up Imaging	Biopsy Proven Metastasis
GD4	γδ MF	E/D	n	T2	Multiple	y	64	PET-CT: no visceral involvement	83	PET-CT: hypermetabolic area in gastric fundus	y
GD5	PCGDTL	E/D	n	T3	Multiple	n	4	PET-CT: no visceral involvement	9	CT: numerous hypo-enhancing lesions within both lobes of the liver	y
GD14	PCGDTL	E/D	n	T3	Multiple	y	1	PET-CT: moderate prominence of uptake in the proximal colon			n
GD21	IVGDTL	IV	n	T1	Multiple	n	0	PET-CT: diffuse metabolic uptake in spleen with splenomegaly	3	PET-CT: diffuse metabolic uptake in spleen with splenomegaly	n
GD26	PCGDTL	E/D	n	T2	Multiple	y	1	CT: thickening of ileal wall portion			n
GD39	PCGDTL	Panniculitic	n	T3	Multiple	n		PET-CT: no visceral involvement		Suspicious for disease spread to liver and spleen	n
GD41	PCGDTL	Panniculitic	n	T3	Multiple	n		PET-CT: no visceral involvement		Suspicious for disease spread to liver, spleen and CNS	n

Supplementary Table 11: Cutaneous disease and imaging timeline in PCGDTL and γδ MF patients with abdominal metastases. Primary cutaneous γδ T cell lymphoma (PCGDTL), epidermal/dermal (E/D), Intravascular (IV), no (n), y (yes), positron emission tomography–computed tomography (PET-CT), computed tomography (CT), central nervous system (CNS).

Uldrich et al, CDR3γ	Uldrich et al, Vγ chain	CGDTL CDR3γ	CGDTL Vγ chain
CATWD**R**LY**Y**KKLF*	TRGV5	CATWD**R**PKA**Y**KKLF*	TRGV5
CATWD**R**GNPKTHY**Y**KKLF	TRGV5	CATWD**R**P**Y**KKLF	TRGV3
CATWD**R**PEANY**Y**KKLF	TRGV5	CATWDSPGKLF	TRGV3
CATWPPYSSDWIKTF	TRGV8	CATWDGSGWIKTF	TRGV2
CATWDGLSYKKLF	TRGV2	CATWDVKGYKKLF	TRGV5
CALWEARPFYYKKLF	TRGV9	CATWDRPMVGKKLF	TRGV3
CATWDALAKLF	TRGV5		

Supplementary Table 12: Published CDR3γ's in Vδ1 cells identified as CD1d-α-galactosylceramide positive in normal human peripheral blood and CDR3γ's from Vδ1 CGDTLs. Bolded are arginine and tyrosine residues identified by crystal structure to be critical for binding to lipid antigen in Uldrich, et al. * indicates TCR tested functionally in this study.

Sample	CNVs	Arm-level	Estimated Purity
GD3	122	19	85.38
GD4	61	6	54.87
GD5	29	1	97.79
GD6	661	8	78.07
GD7	279	5	86.67
GD8*	64	0	93.97
GD9	160	14	89.32
GD10	19	2	94.58
GD12*	71	0	97.79
GD13	149	6	60.45
GD14*	60	0	99.81
GD15	201	10	79.53
GD17*	239	8	99.00
GD34*	173	3	99.00
GD39	547	2	76.77
GD40	868	22	72.24
GD41	563	3	69.34
GD42*	276	3	94.42

Supplementary Table 13: Number of CNVs and arm-level deletions or amplifications detected in CGDTL samples. * indicates samples with CNVs assessed by whole genome sequencing.

Sample	Nonsynonymous mutations
GD3	228
GD4	294
GD5	155
GD6	129
GD7	65
GD8	88
GD9	169
GD10	57
GD12	165
GD13	218
GD14	63
GD15	1971
GD17	99
GD18**	289
GD21**	96
GD22**	32
GD34	143
GD36*	13
GD37*	7
GD39	94
GD40	87
GD41	152
GD42	133

Supplementary Table 14: Number of nonsynonymous mutations detected in each CGDTL sample. * indicates samples which underwent targeted sequencing (see Methods). ** indicates samples with SNVs detected by RNA-seq.

Sample	Chromosome	Position	Reference	Alternate	Distance from *TERT* TSS
GD36	5	1295228	G	A	-124 bp
GD4	5	1295250	G	A	-146 bp

Supplementary Table 15: *TERT* promoter mutations in CGDTL. TSS (Transcriptional start site).

Cytoband	Q value	Residual Q value	Chr	Start	End	Number of genes	DEL/AMP
9p21.3	3.17E-14	3.17E-14	9	21838260	23693134	6	DEL
16p13.3	0.0077873	0.013554	16	1252703	1665235	16	DEL
1p13.2	0.029978	0.029978	1	113934222	115534150	18	DEL
4q35.2	0.049309	0.049309	4	189196352	191154276	9	DEL
1p36.11	0.07195	0.07195	1	26601813	28362440	42	DEL
9q21.33	0.090527	0.090527	9	86660872	91150870	24	DEL
1p36.32	0.10855	0.10855	1	3409582	3774530	10	DEL
7p22.3	0.10855	0.10855	7	1	2611537	41	DEL
1p34.3	0.19059	0.19059	1	39464234	40254406	12	DEL
16p13.13	0.099517	0.2441	16	10840197	13001595	22	DEL
15q11.2	2.32E-12	2.32E-12	15	1	22083019	14	AMP
16p11.2	0.00015499	0.00015499	16	31392579	31428216	2	AMP
2q12.2	0.00071935	0.00071935	2	85788309	108609832	172	AMP
1q23.3	0.0011043	0.0011043	1	159904169	161163669	42	AMP
1p36.33	0.0039872	0.0039872	1	912312	977791	4	AMP
1p36.22	0.024196	0.024196	1	11851110	15713565	53	AMP
6q23.2	0.099361	0.099361	6	131215832	131520962	2	AMP
8q13.1	0.099361	0.099361	8	67357219	67579442	4	AMP
11p15.5	0.099361	0.099361	11	410278	433979	2	AMP
17p13.1	0.099361	0.099361	17	5429997	7111807	33	AMP
1p35.2	0.14252	0.14252	1	29525762	32263555	26	AMP
12q24.12	0.14252	0.14252	12	111873082	112242026	6	AMP
6p21.1	0.15101	0.15101	6	42982089	44281340	40	AMP
17q24.2	0.17686	0.17686	17	65750982	66597056	12	AMP
22q12.3	0.193	0.193	22	36424724	36717190	6	AMP
17q12	0.19888	0.19888	17	35748929	36862243	22	AMP
7q36.1	0.24889	0.24889	7	150715260	159138663	63	AMP

Supplementary Table 16: Significant deletions and amplifications identified using GISTIC2.0. Chromosome (Chr), deletion (DEL), amplification (AMP).

Gene	γδ MF without PCGDTL-like Progression (n=2)	γδ MF with PCGDTL-like progression (n=4)	E/D PCGDTL (n=6)	*P* Value
KRAS			2	0.4545
MAPK1			2	0.4545
TP53	1	3	1	1
JAK3		1		1
CDKN2A		5	4	1
MYC		1		1
ARID1A	1	3	3	1
TNFAIP3		1	1	1
TRRAP		1	1	1
KMT2D	1	2	2	1
TET2	1		1	1
FBXW7		1	1	1
STAT5B			2	0.4545

Supplementary Table 17: Frequency of driver mutations in Vδ1 γδ MF and PCGDTL. *P* Value determined via Fisher's Exact Test, comparing Vδ1 γδ MF to Vδ1 E/D PCGDTL.

Gene	Vδ1 PCGDTL and γδ MF	Vδ2 PCGDTL	*P* Value
KRAS	2	2	1
MAPK1	2	0	0.4857
TP53	5	2	0.6424
JAK3	1	0	1
CDKN2A	9	3	0.0872
MYCN	0	3	0.0632
MYC	1	1	1
ARID1A	7	4	0.6699
STAT3	0	1	0.4286
TNFAIP3	2	1	1
TRRAP	2	1	1
KMT2D	5	2	0.6424
TET2	2	0	0.4857
FBXW7	2	2	1
NRAS	0	1	0.4286

Supplementary Table 18: Frequency of driver mutations in PCGDTL and γδ MF by cell of origin. *P* Value determined via Fisher's Exact Test, comparing Vδ1 γδ MF and Vδ1 E/D PCGDTL to Vδ2 PCGDTL.

	γδ MF	γδ MF with PCGDTL- like progression	E/D PCGDTL	Panniculitic PCGDTL
Current WHO-EORTC Classification	Variant of Mycosis Fungoides	Variant of Mycosis Fungoides	PCGDTL	PCGDTL
Suggested Revised Classification	Vδ1 PCGDTL	Vδ1 PCGDTL	Vδ1 PCGDTL	Vδ2 PCGDTL

Supplementary Table 19: WHO Subtypes of Cutaneous γδ T cell Lymphomas and Suggested Reclassification. Epidermal/Dermal (E/D), Primary cutaneous gamma delta T cell Lymphoma (PCGDTL).
