# Supplementary material for: Cellular origins and genetic landscape of cutaneous gamma delta T cell lymphomas
Source: Nat Commun. 2020 Apr 14;11:1806. doi: 10.1038/s41467-020-15572-7 (PMC7156460; doi:10.1038/s41467-020-15572-7)
Supplement: Supplementary file 2 — Description of Additional Supplementary Information [file 41467_2020_15572_MOESM2_ESM.pdf]

Description of Additional Supplementary Files

File Name: Supplementary Data 1
Description: Genes differentially expressed between Vδ1 and Vδ2 lymphomas. DESeq2 results comparing Vδ1 and Vδ2 samples. Negative Log2FC indicates genes more highly expressed in Vδ1 lymphomas, and positive Log2FC indicates genes more highly expressed in Vδ2 lymphomas.

File Name: Supplementary Data 2
Description: Somatic single nucleotide variants detected in CGDTLs.

File Name: Supplementary Data 3
Description: Arm level events detected in PCGDTL and γδ MF cases.

File Name: Supplementary Data 4
Description: Driver mutations, treatments and patient outcomes in PCGDTL and γδ MF.
