# Supplementary material for: Cellular origins and genetic landscape of cutaneous gamma delta T cell lymphomas
Source: Nat Commun. 2020 Apr 14;11:1806. doi: 10.1038/s41467-020-15572-7 (PMC7156460; doi:10.1038/s41467-020-15572-7)
Supplement: Supplementary file 7 — Reporting Summary [file 41467_2020_15572_MOESM7_ESM.pdf]

nature research

Corresponding author(s): Abner Louissaint, Joan Guitart, Jaehyuk Choi

Last updated by author(s): Feb 7, 2020

Reporting Summary

Nature Research wishes to improve the reproducibility of the work that we publish. This form provides structure for consistency and transparency in reporting. For further information on Nature Research policies, see Authors & Referees and the Editorial Policy Checklist .

Statistics

For all statistical analyses, confirm that the following items are present in the figure legend, table legend, main text, or Methods section.

n/a	Confirmed	
☐	☒	The exact sample size (*n*) for each experimental group/condition, given as a discrete number and unit of measurement
☐	☒	A statement on whether measurements were taken from distinct samples or whether the same sample was measured repeatedly
☐	☒	The statistical test(s) used AND whether they are one- or two-sided<br>*Only common tests should be described solely by name; describe more complex techniques in the Methods section.*
☐	☒	A description of all covariates tested
☐	☒	A description of any assumptions or corrections, such as tests of normality and adjustment for multiple comparisons
☐	☒	A full description of the statistical parameters including central tendency (e.g. means) or other basic estimates (e.g. regression coefficient) AND variation (e.g. standard deviation) or associated estimates of uncertainty (e.g. confidence intervals)
☐	☒	For null hypothesis testing, the test statistic (e.g. *F*, *t*, *r*) with confidence intervals, effect sizes, degrees of freedom and *P* value noted<br>*Give P values as exact values whenever suitable.*
☒	☐	For Bayesian analysis, information on the choice of priors and Markov chain Monte Carlo settings
☒	☐	For hierarchical and complex designs, identification of the appropriate level for tests and full reporting of outcomes
☒	☐	Estimates of effect sizes (e.g. Cohen's *d*, Pearson's *r*), indicating how they were calculated

Our web collection on statistics for biologists contains articles on many of the points above.

Software and code

Policy information about availability of computer code

Data collection	BDFacsDiva version 8.0
Data analysis	R version 3.5.1<br>Python version 2.7.13<br>Bwa version 0.7.12<br>samtools version 1.6<br>Picard version 1.131<br>MuTect version 1.1.7<br>GATK 4.0.0<br>GISTIC2.0 version 2.0.23<br>SOBDetector version 0.1<br>Patchwork version 2.4<br>MuSiCa version 1.0<br>STAR version 2.6.0a<br>HTSeq version 0.6.0<br>DeSEQ2 version 1.10.1<br>Ballgown version 2.16.0<br>PRADA version 1.2<br>GSVA version 1.18.0<br>ChEA accessed at http://amp.pharm.mssm.edu/Enrichr/<br>MiXCR version 2.1.10<br>GraphPad Prism version 8.0.0<br>ImmunoSeq Analyzer version 3.0

FlowJo version 10.5.3
Modeller version 9v8
Rosetta version 3.2

For manuscripts utilizing custom algorithms or software that are central to the research but not yet described in published literature, software must be made available to editors/reviewers. We strongly encourage code deposition in a community repository (e.g. GitHub). See the Nature Research guidelines for submitting code & software for further information.

Data

Policy information about availability of data

All manuscripts must include a data availability statement. This statement should provide the following information, where applicable:

- Accession codes, unique identifiers, or web links for publicly available datasets
- A list of figures that have associated raw data
- A description of any restrictions on data availability

A data availability statement is provided in the manuscript.

Field-specific reporting

Please select the one below that is the best fit for your research. If you are not sure, read the appropriate sections before making your selection.

[x] Life sciences [ ] Behavioural & social sciences [ ] Ecological, evolutionary & environmental sciences

For a reference copy of the document with all sections, see nature.com/documents/nr-reporting-summary-flat.pdf

Life sciences study design

All studies must disclose on these points even when the disclosure is negative.

Sample size	Because primary cutaneous gamma delta T cell lymphoma is rare and available tissue is limited, we did not predetermine sample sizes.
Data exclusions	We excluded one patient who did not have evidence of gamma-delta T cell receptor expression. This patient was originally diagnosed with primary cutaneous gamma delta T cell lymphoma but was excluded due to positive alpha-beta T cell receptor and negative gamma-delta T cell receptor staining by immunohistochemistry.
Replication	CD1d lipid binding experiments in HEK293 cells were replicated across 3 independent experiments. Flow cytometry results from normal human epidermis and dermis were similar across donors tested (n=5). Analysis of the cell of origin by T cell receptor sequencing based on samples from Northwestern Memorial Hospital were replicated when attempted with samples from Massachusetts General Hospital.
Randomization	Samples in our study were not randomized into experimental groups. We performed sequencing of samples on the basis of available tissue.
Blinding	Analysis of next generation sequencing data was blinded to clinical outcomes. In other experiments, researchers were not blinded during data collection or analysis.

Reporting for specific materials, systems and methods

We require information from authors about some types of materials, experimental systems and methods used in many studies. Here, indicate whether each material, system or method listed is relevant to your study. If you are not sure if a list item applies to your research, read the appropriate section before selecting a response.

Materials & experimental systems

n/a	Involved in the study	
[ ]	[x]	Antibodies
[x]	[ ]	Eukaryotic cell lines
[x]	[ ]	Palaeontology
[x]	[ ]	Animals and other organisms
[ ]	[x]	Human research participants
[ ]	[x]	Clinical data

Methods

n/a	Involved in the study	
[x]	[ ]	ChIP-seq
[ ]	[x]	Flow cytometry
[x]	[ ]	MRI-based neuroimaging

Antibodies

Antibodies used	PB-CD3, Biolegend, Clone OKT3<br>PB-Vδ1, Miltenyi Biotech, Clone REA173

	PE-Vδ2, Miltenyi Biotech, Clone 123R3<br>FITC-γδTCR, Invitrogen, Clone 5A6.E9<br>APC-Vγ9, Biolegend, Clone B3<br>APC-CD1d-PBS-57 tetramer, NIH Tetramer Core Factility<br>TCR C gamma M1, ThermoFisher Scientific, Clone γ3.20<br>TCR δ, Santa Cruz Biotechnology, Clone H-41
Validation	All antibodies were validated by the manufacturer.

Human research participants

Policy information about studies involving human research participants

Population characteristics	Characteristics of patients with cutaneous gamma-delta T cell lymphoma studied including age, gender, and race when known are presented in Table S1.
Recruitment	Patients with cutaneous gamma-delta T cell lymphoma with annotated clinical information and/or tissue sufficient for sequencing analysis were included. Patients were recruited through cutaneous lymphoma clinic and provided informed consent for the collection of tumor samples for research purposes.
Ethics oversight	This study was approved by the institutional review boards of Northwestern University, Harvard University, University of Virginia, and University of Chicago.

Note that full information on the approval of the study protocol must also be provided in the manuscript.

Clinical data

Policy information about clinical studies

All manuscripts should comply with the ICMJEguidelines for publication of clinical research and a completedCONSORT checklist must be included with all submissions.

Clinical trial registration	Although this study includes clinical data, this study is not a clinical trial.
Study protocol	Clinical trial study protocols were not utilized. Ethical approval for this study was obtained from institutional review boards.
Data collection	All samples at participating institutions with a clinical diagnosis of cutaneous lymphomas, immunohistochemical staining or next generations sequencing evidence of gamma-delta T cell origin, and who provided informed consent were included. Samples with sufficient tissue for sequencing analysis were utilized for sequencing studies. Clinical data was obtained from the electronic medical records of each patient, when available.
Outcomes	We did not predefine outcomes in this study as in a clinical trial.

Flow Cytometry

Plots

Confirm that:

- [x] The axis labels state the marker and fluorochrome used (e.g. CD4-FITC).
- [x] The axis scales are clearly visible. Include numbers along axes only for bottom left plot of group (a 'group' is an analysis of identical markers).
- [x] All plots are contour plots with outliers or pseudocolor plots.
- [x] A numerical value for number of cells or percentage (with statistics) is provided.

Methodology

Sample preparation	Detailed descriptions of the strategy for mononuclear cell isolation from human skin and flow cytometry are provided in the Methods. Skin from healthy donors undergoing abdominoplasty was obtained, subcutaneous tissue seperated, and digestion performed overnight in 60% RPMI/40% dispase. Epidermis and dermis were seperated, and all samples were filtered by cell straining. Mononuclear cells were then isolated by density centrifugation. For all flow cytometry experiments, single cell suspensions were generated by resuspending cell pellets with phosphate buffered saline/2% fetal bovine serum. Cells were stained with CD1d-PBS-57 tetramers, with incubation at room temperature for 30 minutes protected from light. Cells were subsequently stained with cell surface antibodies for 20 minutes at room temperature protected from light. Cells were then washed with phosphate buffered saline/2% fetal bovine serum.
Instrument	Flow cytometry data was collected on a BD LSR II machine (BD Biosciences), and sorting was performed on a BD FACSARIA 5 (BD Bioscience).
Software	Flow cytometry data was collected using BDFACSDiva software (version 8.0) and was analyzed using FlowJo Software (version v10.5.3).

Cell population abundance	Purity of γδTCR transduced cells was verified by flow cytometry to be >80% pure following 2 days of expansion. Purity of tumor cells isolated for scRNA-seq was assessed via scRNA sequencing and confirmed >99% of TCR delta reads supporting the malignant clonotype.
Gating strategy	The gating strategy for identifiying γδ T cells in normal human skin is shown in Figure S5. The gating strategy for CD1d-lipid binding experiments is shown in Figure S6.

☒ Tick this box to confirm that a figure exemplifying the gating strategy is provided in the Supplementary Information.
